# Supplementary material for: Differential relationship between meditation methods and psychotic-like and mystical experiences
Source: PLoS One. 2024 Dec 5;19(12):e0309357. doi: 10.1371/journal.pone.0309357 (PMC11620446; doi:10.1371/journal.pone.0309357)
Supplement: S1 File — (DOCX) [file pone.0309357.s001.docx]

**S1**

*List of Meditation Techniques*

(1) Scanning the entire body

(2) Being mindful of the rise and fall of the abdomen while breathing

(3) Observing how thoughts arise in the mind without adhering to them

(4) Being mindful of the respiratory flow in the entire body

(5) Perceiving, then releasing emotions and tensions (e.g., with the help of the breath), while scanning the body

(6) Cultivating compassion, sympathetic joy, equanimity, loving kindness (for oneself, friends, neutral people, enemies, the whole world)

(7) Observing how bodily sensations arise without adhering to them

(8) Singing sutras/mantras

(9) Being mindful of the sensations arising in the nose during inhalation and exhalation

(10) Lying down and going into a state of deep relaxation while being fully conscious

(11) Accumulating energy in specific centers (e.g., abdomen) and channeling it through certain pathways (e.g., spine)

(12) Carrying out predetermined, meditative sequences of movements while allowing the breath to flow naturally

(13) Combining inhalation and exhalation with visualization of energy, qualities, light, smoke, etc.

(14) Concentrating on a location in the body (e.g., abdomen or an "energy center" like chakra, Dan Tien) or on a series of locations in the body/"energy centers"

(15) Concentrating the mind on something contradictory without thinking about the contradiction

(16) Contemplating a spiritually important question (e.g., "Who am I?")

(17) Contemplating death and one's own mortality

(18) Contemplating the conditional emergence of experiences (cause & effect)

(19) Counting breaths

(20) Creating a visual representation of a deity and then merging with this visualization

(21) Droning or humming continuously with optional corresponding hand movements

(22) Fixating on an object without blinking/"staring" (candle flame, picture, hand)

(23) Focusing on internal sounds and vibrations

(24) Focusing on one point of the body and letting the breath flow through this point of concentration

(25) Focusing on the pauses between inhalation and exhalation, carefully observing what happens

(26) Fostering and focusing on a spiritual connection created by singing together

(27) Labeling mental experiences with words that describe these experiences

(28) Listening to the sound of singing bowls or a gong and feeling the corresponding vibrations inside the body

(29) Looking at/focusing on a sacred object (image of the master, sacred geometric pattern, etc.)

(30) Mentally repeating syllables or words while connecting them to the rhythm of breathing

(31) Observing emotions without adhering to them

(32) Opening oneself up to blessings and inspiration

(33) Reading certain paragraphs of a text over and over again and taking them in

(34) Reciting a mantra loudly, in a whisper, and silently

(35) Repeating a mantra using a mnemonic (e.g., prayer beads)

(36) Repeating a mantra while focusing on corresponding points in the body

(37) Repeating an affirmation (e.g., "I am patient")

(38) Sitting and gazing at the wall, observing oneself doing nothing

(39) Sitting or standing upright with the eyes closed and allowing the body to move smoothly without intervening

(40) Spinning around one's own axis with the arms spread out

(41)Trying to feel one's heartbeat

(42) Visualizations associated with light or fire at different body parts

(43) Visualizations associated with the opening of the heart (e.g., rose blossom)

(44) Visualizing how the dead human body slowly decays and decomposes

(45) Visualizing that thoughts are inherently restless and focusing on the silence and the vastness that lies beyond them

(46) Visualizing the body expanding in all directions

(47) Voluntary manipulation of breath, e.g., reducing the strength of breathing or "pranayama" with holding one's breath

(48) Walking and being mindful of sensory perceptions (movement of the feet, legs, clothing, air, hair etc.), coordinating it with the breath if necessary

(49) Walking, dividing the walking process into parts, and internally labeling each partial movement

(50) With a specific intention (e.g., open one's heart, raise one's mood) selecting and repeating a mantra, combining it with associated hand postures or arm movements

**S2**

| *Meditation Traditions* |  |
| --- | --- |
| No tradition/free meditation | Tantra |
| Mindfulness-based stress reduction | Shameanism |
| Mindfulness Based Cognitive Therapy | Anthoposophy |
| Meditation App | Merkaba/Jewish |
| Theravada, Vipassana | Vaishhavism |
| Tibetan Buddhism | Sri Chinmoy |
| Sufism | Sri Aurobindo |
| Christianity | Mother Meera |
| Zen | Ramana Maharshi |
| Sivananda Yoga | Deepak Chopra |
| Yoga (other) | Transcendental Meditation |
| Kundalini Yoga | Acem |
| Osho meditation | Other |
| Qigong/Tai Chi | Tantra |
| No tradition/free meditation | Shameanism |
| Mindfulness-based stress reduction | Anthoposophy |
| Mindfulness Based Cognitive Therapy | Merkaba/Jewish |
| Meditation App | Vaishhavism |
| Theravada, Vipassana | Sri Chinmoy |
| Tibetan Buddhism | Sri Aurobindo |
| Sufism | Mother Meera |
| Christianity | Ramana Maharshi |
| Zen | Deepak Chopra |
| Sivananda Yoga | Transcendental Meditation |
| Yoga (other) | Acem |
| Kundalini Yoga | Other |
| Osho meditation |  |
| Qigong/Tai Chi |  |

**S 3**

*Reasons to Meditate Within Each Factor*

| **Factor 1 Reasons (SpiritExplore)** | **Factor 2 Reasons (Health)** |
| --- | --- |
| (2) Self-exploration (e.g. Explore not yet conscious aspects of my mind, Understand how my mind works) | (1) Calm down/feel better (e.g. Calm down internally, Relax, Find good work–life balance, Become more serene) |
| (5) Seek own spiritual experience (e.g. Connect to a higher power, Experience altered states of consciousness) | (3) Reaction to problems (e.g. Beca I am feeling unhappy in my present situation, Beca I want to change my way of life) |
| (12) Developing paranormal/psychic powers | (4) Open up (e.g. Open my heart, Accept myself, Direct my awareness to the present moment) |
| (13) To move towards a non-dual state of emptiness or oneness during a meditative state (Other terms: Buddha Consciousness, cosmic consciousness, pure consciousness, true-Self, non-Self) | (6) Connecting with nature/self-caring (e.g. Be more aware of my body, Consciously perceive beauty of nature, Mindfulness ) |
| (14) Liberation/Seek enlightenment | (7) Compassion (e.g. Have more compassion for others, contribute to a change in society by meditating) |
|  | (8) Mental improvement (e.g. Improve my intellectual capabilities, Improve my concentration, Become more creative) |
|  | (9) Deal better with problems (e.g. Deal better with grief, Learn to deal better with pain) |
|  | (10) Personal development (e.g. Recognize and remove negative thoughts and beliefs, Make my mind familiar with new positive thoughts and images) |
|  | (11) Contentment and clarity (e.g. Experience deep contentment and inner happiness in the present, Experience inner clarity) |

| **S 4** | | |
| --- | --- | --- |
| *Benjamini-Hochberg FDR Procedure for Pre-registered Hypotheses* | | |
| Pre.registered.Hypothesis | *p* | adjusted_p_value |
| Hyp 1.a | < .001*** | < .001*** |
| Hyp 1. c | < .001*** | < .001*** |
| Hyp 2. a | < .001*** | < .001*** |
| Hyp 3. a | < .001*** | < .001*** |
| Hyp 4. a | < .001*** | < .001*** |

*Note.* * p < .05, ** p < .01, *** p < .001

| **S 5** | | | | | |
| --- | --- | --- | --- | --- | --- |
| *Meditation Category and PLEs in Daily Life* | | | | | |
| Term | ß | *SE* | *t* | *p* | 95% CI |
| (Intercept) | 40.06 | 1.21 | 33.07 | < .001 | [37.68, 42.43]*** |
| NDM | 0.39 | 0.05 | 8.00 | < .001 | [0.30, 0.49]*** |
| CDM | -0.07 | 0.03 | -2.30 | .022 | [-0.13, -0.01]* |
| ADM | 0.02 | 0.05 | 0.35 | .724 | [-0.08, 0.11] |
| Age | -0.19 | 0.02 | -8.46 | < .001 | [-0.24, -0.15]*** |
| Sex: Female Vs. Male | -0.16 | 0.64 | -0.25 | .804 | [-1.43, 1.11] |
| Sex: Prefer Not to Say Vs. Male | -1.82 | 3.84 | -0.47 | .637 | [-9.36, 5.73] |
| *Note.* * p < .05, ** p < .01, *** p < .001 | | | | | |
|  | | | | | |

| **S 6** | | | | |
| --- | --- | --- | --- | --- |
| *Partial Eta-squared Effect Sizes for Regression Analyses* | | | | |
| Hypothesis and Variable | Effect Size | CI | CI Low | CI High |
| Hyp 1). a) NDM | 0.10 | 0.95 | 0.06 | 1.00 |
| Hyp 1). a) CDM | 0.00 | 0.95 | 0.00 | 1.00 |
| Hyp 1). a) ADM | 0.00 | 0.95 | 0.00 | 1.00 |
| Hyp 1). a) Age | 0.10 | 0.95 | 0.07 | 1.00 |
| Hyp 1). a) Sex | 0.00 | 0.95 | 0.00 | 1.00 |
| Hyp 1). c) Med Category | 0.02 | 0.95 | 0.01 | 1.00 |
| Hyp 1). c) Age | 0.00 | 0.95 | 0.00 | 1.00 |
| Hyp 1). c) Sex | 0.00 | 0.95 | 0.00 | 1.00 |
| Hyp 2). a) Retreat Days | 0.00 | 0.95 | 0.00 | 1.00 |
| Hyp 2). a) Med Months | 0.01 | 0.95 | 0.00 | 1.00 |
| Hyp 2). a) Med Hours Wk | 0.00 | 0.95 | 0.00 | 1.00 |
| Hyp 2). a) Med Reg | 0.01 | 0.95 | 0.00 | 1.00 |
| Hyp 2). a) Age | 0.06 | 0.95 | 0.03 | 1.00 |
| Hyp 2). a) Sex | 0.00 | 0.95 | 0.00 | 1.00 |
| Hyp 3). a) Model 1 NDM | 0.09 | 0.95 | 0.03 | 1.00 |
| Hyp 3). a) Model 1 CDM | 0.01 | 0.95 | 0.00 | 1.00 |
| Hyp 3). a) Model 1 ADM | 0.00 | 0.95 | 0.00 | 1.00 |
| Hyp 3). a) Model 1 Nationality | 0.01 | 0.95 | 0.00 | 1.00 |
| Hyp 3). a) Model 1 Sleep | 0.10 | 0.95 | 0.03 | 1.00 |
| Hyp 3). a) Model 1 DAS | 0.19 | 0.95 | 0.10 | 1.00 |
| Hyp 3). a) Model 1 ASI | 0.09 | 0.95 | 0.02 | 1.00 |
| Hyp 3). a) Model 1 Trauma | 0.00 | 0.95 | 0.00 | 1.00 |
| Hyp 3). a) Model 1 PsychFam | 0.02 | 0.95 | 0.00 | 1.00 |
| Hyp 3). a) Model 1 Age | 0.06 | 0.95 | 0.01 | 1.00 |
| Hyp 3). a) Model 1 Sex | 0.01 | 0.95 | 0.00 | 1.00 |
| Hyp 3). a) Model 1 NDM × Nationality | 0.00 | 0.95 | 0.00 | 1.00 |
| Hyp 3). a) Model 1 NDM × Sleep | 0.00 | 0.95 | 0.00 | 1.00 |
| Hyp 3). a) Model 1 NDM × DAS | 0.02 | 0.95 | 0.00 | 1.00 |
| Hyp 3). a) Model 1 NDM × ASI | 0.01 | 0.95 | 0.00 | 1.00 |
| Hyp 3). a) Model 1 NDM × Trauma | 0.00 | 0.95 | 0.00 | 1.00 |
| Hyp 3). a) Model 1 NDM × PsychFam | 0.04 | 0.95 | 0.00 | 1.00 |
| Hyp 3). a) Model 1 CDM × Nationality | 0.01 | 0.95 | 0.00 | 1.00 |
| Hyp 3). a) Model 1 CDM × Sleep | 0.00 | 0.95 | 0.00 | 1.00 |
| Hyp 3). a) Model 1 CDM×DAS | 0.06 | 0.95 | 0.01 | 1.00 |
| Hyp 3). a) Model 1 CDM × ASI | 0.07 | 0.95 | 0.01 | 1.00 |
| Hyp 3). a) Model 1 CDM × Trauma | 0.00 | 0.95 | 0.00 | 1.00 |
| Hyp 3). a) Model 1 CDM × PsychFam | 0.01 | 0.95 | 0.00 | 1.00 |
| Hyp 3). a) Model 1 ADM × Nationality | 0.04 | 0.95 | 0.00 | 1.00 |
| Hyp 3). a) Model 1 ADM × Sleep | 0.00 | 0.95 | 0.00 | 1.00 |
| Hyp 3). a) Model 1 ADM × DAS | 0.01 | 0.95 | 0.00 | 1.00 |
| Hyp 3). a) Model 1 ADM × ASI | 0.01 | 0.95 | 0.00 | 1.00 |
| Hyp 3). a) Model 1 ADM × Trauma | 0.00 | 0.95 | 0.00 | 1.00 |
| Hyp 3). a) Model 1 ADM × PsychFam | 0.01 | 0.95 | 0.00 | 1.00 |
| Hyp 4). a) Mystical | 0.05 | 0.95 | 0.01 | 1.00 |
| Hyp 4). a) Age | 0.08 | 0.95 | 0.03 | 1.00 |
| Hyp 4). a) Sex | 0.01 | 0.95 | 0.00 | 1.00 |

________________________________________________________________

| **S 7** | | | | | | |
| --- | --- | --- | --- | --- | --- | --- |
| *Difference from Zero of Meditation Methods Likelihood Ratings for PLEs In Daily Life* | | | | | | |
| Meditation Category | *n* | Mean | *SD* | *df* | *t* | p-value |
| NDM | 613 | -0.42 | 4.75 | 612 | 2.19 | 0.05* |
| CDM | 613 | -3.25 | 10.94 | 612 | -7.35 | <.0.001*** |
| ADM | 613 | -3.02 | 10.12 | 612 | -7.38 | <.0.001*** |
| *Note.* * p < .05, ** p < .01, *** p < .001 | | | | | | |
|  | | | | | | |

| **S 8** | | | | | |
| --- | --- | --- | --- | --- | --- |
| *Meditation Method Contrasts and Likelihood of PLEs in Daily Life* | | | | | |
| contrasts.contrats | contrasts.estimate | contrasts.SE | contrasts.df | contrasts.t.ratio | contrasts.p.value |
| ADM-CDM | 0.23 | 0.52 | 1,836 | 0.44 | 0.90 |
| ADM-NDM | -2.60 | 0.52 | 1,836 | -5.04 | <.0.001*** |
| CDM-NDM | -2.83 | 0.52 | 1,836 | -5.48 | <.0.001*** |
|  |  |  |  |  |  |
| *Note.* * p < .05, ** p < .01, *** p < .001 | | | | | |
|  | | | | | |
|  | | | | | |

| **S 9** | | | | | |  |
| --- | --- | --- | --- | --- | --- | --- |
| *Meditation Method Contrasts and Likelihood of PLEs in Daily Life PLE Experiencers Only* | | | | | |  |
| Contrast | *contrasts.estimate* | *SE* | *df* | *contrasts.t.ratio* | *contrasts.p.value* | p_value |
| ADM - CDM | 0.25 | 0.52 | 1,818 | 0.47 | 0.88 | <0.05* |
| ADM - NDM | -2.52 | 0.52 | 1,818 | -4.86 | <0.001*** | <0.001*** |
| CDM - NDM | -2.76 | 0.52 | 1,818 | -5.33 | <0.001*** | <0.001*** |

*Note.* * p < .05, ** p < .01, *** p < .001

**S 10**

| *Perceived Causality Meditation Methods from Zero in PLE Experiencers Only* | | | | | | |
| --- | --- | --- | --- | --- | --- | --- |
| Meditation.Category | *n* | Mean | *SD* | *df* | *t* | p_value |
| NDM | 607 | -0.43 | 4.77 | 606 | -2.24 | <0.05* |
| CDM | 607 | -3.19 | 10.93 | 606 | -7.21 | <0.001*** |
| ADM | 607 | -2.95 | 10.12 | 606 | -7.21 | <0.001*** |

*Note.* * p < .05, ** p < .01, *** p < .001

| **S 11** | | | | | |
| --- | --- | --- | --- | --- | --- |
| *PLEs Predicted by Meditation Total Habits* | | | | | |
| Term | ß | *SE* | *t* | *p* | 95% CI |
| (Intercept) | 41.06 | 1.22 | 33.54 | < .001*** | [38.66, 43.47] |
| Retreat Days | 0.00 | 0.00 | 1.10 | .271 | [-0.00, 0.00] |
| Med Months | -0.00 | 0.00 | -0.01 | .991 | [-0.01, 0.01] |
| Med Hours Wk. | 0.03 | 0.04 | 0.75 | .452 | [-0.04, 0.10] |
| Med Reg | -0.24 | 0.14 | -1.78 | .075 | [-0.51, 0.02] |
| Age | -0.16 | 0.03 | -6.03 | < .001*** | [-0.21, -0.11] |
| Sex2 | -0.57 | 0.67 | -0.84 | .399 | [-1.89, 0.75] |
| Sex3 | -0.19 | 4.09 | -0.05 | .964 | [-8.22, 7.85] |

*Note.* * p < .05, ** p < .01, *** p < .001

| **S 12** | | | | | |
| --- | --- | --- | --- | --- | --- |
| *Effects of Psychosis Proneness on Meditation and PLEs* | | | | | |
| Term | ß | *SE* | *t* | *p* | 95% CI |
| (Intercept) | 31.22 | 5.25 | 5.95 | < .001*** | [20.84, 41.61] |
| NDM | 0.15 | 0.35 | 0.43 | .671 | [-0.54, 0.84] |
| CDM | 0.06 | 0.21 | 0.29 | .772 | [-0.35, 0.48] |
| ADM | -0.34 | 0.30 | -1.11 | .269 | [-0.94, 0.26] |
| Nationality | -1.81 | 1.23 | -1.48 | .142 | [-4.24, 0.61] |
| Sleep | -0.04 | 0.18 | -0.23 | .815 | [-0.39, 0.31] |
| DAS | 0.39 | 0.10 | 3.80 | < .001*** | [0.18, 0.59] |
| ASI | -0.19 | 0.09 | -2.14 | .035* | [-0.36, -0.01] |
| Trauma | 0.45 | 0.45 | 0.99 | .327 | [-0.45, 1.35] |
| Schiz | 4.80 | 2.95 | 1.63 | .106 | [-1.04, 10.64] |
| Age | -0.07 | 0.03 | -2.45 | .016* | [-0.13, -0.01] |
| Sex | 0.75 | 0.95 | 0.78 | .434 | [-1.14, 2.63] |
| NDM × Nationality | 0.13 | 0.08 | 1.64 | .104 | [-0.03, 0.28] |
| NDM × Sleep | -0.01 | 0.01 | -0.55 | .584 | [-0.03, 0.02] |
| NDM × DAS | -0.00 | 0.01 | -0.40 | .690 | [-0.02, 0.01] |
| NDM × ASI | 0.01 | 0.01 | 0.97 | .333 | [-0.01, 0.02] |
| NDM × Trauma | -0.03 | 0.03 | -0.92 | .361 | [-0.10, 0.03] |
| NDM × Schiz | 0.40 | 0.16 | 2.58 | .011* | [0.09, 0.71] |
| CDM × Nationality | 0.16 | 0.06 | 2.77 | .006** | [0.05, 0.27] |
| CDM × Sleep | -0.01 | 0.01 | -0.99 | .324 | [-0.02, 0.01] |
| CDM × DAS | -0.01 | 0.00 | -3.47 | .001*** | [-0.02, -0.01] |
| CDM × ASI | 0.01 | 0.00 | 2.63 | .010** | [0.00, 0.02] |
| CDM × Trauma | -0.01 | 0.02 | -0.77 | .441 | [-0.05, 0.02] |
| CDM × Schiz | -0.15 | 0.10 | -1.57 | .120 | [-0.34, 0.04] |
| ADM × Nationality | -0.16 | 0.07 | -2.18 | .031* | [-0.30, -0.01] |
| ADM × Sleep | 0.01 | 0.01 | 0.86 | .392 | [-0.01, 0.03] |
| ADM × DAS | 0.00 | 0.01 | 0.41 | .682 | [-0.01, 0.01] |
| ADM × ASI | 0.00 | 0.00 | 1.13 | .262 | [-0.00, 0.01] |
| ADM × Trauma | 0.02 | 0.03 | 0.54 | .593 | [-0.04, 0.07] |
| ADM × Schiz | -0.16 | 0.16 | -1.01 | .314 | [-0.49, 0.16] |

*Note.* * p < .05, ** p < .01, *** p < .001

| **S 13** | | | | | |
| --- | --- | --- | --- | --- | --- |
| *Psychosis Proneness Meditation and PLEs* | | | | | |
| Term | ß | *SE* | *t* | *p* | 95% CI |
| (Intercept) | 28.93 | 2.98 | 9.70 | < .001*** | [23.03, 34.83] |
| NDM | 0.22 | 0.08 | 2.79 | .006** | [0.07, 0.38] |
| CDM | 0.03 | 0.05 | 0.65 | .516 | [-0.06, 0.12] |
| ADM | -0.06 | 0.07 | -0.93 | .353 | [-0.20, 0.07] |
| Nationality | 0.54 | 0.45 | 1.20 | .232 | [-0.35, 1.42] |
| Sleep | -0.13 | 0.06 | -2.22 | .028* | [-0.25, -0.01] |
| DAS | 0.08 | 0.04 | 2.10 | .038* | [0.00, 0.15] |
| ASI | 0.08 | 0.03 | 2.64 | .009** | [0.02, 0.14] |
| Trauma | 0.17 | 0.17 | 0.95 | .343 | [-0.18, 0.51] |
| Schiz | 0.71 | 0.60 | 1.18 | .241 | [-0.48, 1.90] |
| Age | -0.08 | 0.03 | -2.65 | .009** | [-0.14, -0.02] |
| Sex | 0.93 | 0.90 | 1.03 | .306 | [-0.85, 2.71] |

*Note.* * p < .05, ** p < .01, *** p < .001

| **S 14** | | | | | |
| --- | --- | --- | --- | --- | --- |
| *Mystical Experiences and Meditation in Daily Life* | | | | | |
| Term | ß | *SE* | *t* | *p* | 95% CI |
| (Intercept) | 9.93 | 11.47 | 0.87 | .388 | [-12.74, 32.60] |
| NDM | 1.91 | 0.39 | 4.85 | < .001 | [1.13, 2.68]*** |
| CDM | -0.20 | 0.23 | -0.87 | .385 | [-0.67, 0.26] |
| ADM | 0.51 | 0.34 | 1.51 | .133 | [-0.16, 1.19] |
| Age | 0.22 | 0.15 | 1.46 | .145 | [-0.08, 0.52] |
| Sex | 6.15 | 4.38 | 1.40 | .163 | [-2.51, 14.81] |
| *Note.* * p < .05, ** p < .01, *** p < .001 | | | | | |
|  | | | | | |

| **S 15** | | | | | |
| --- | --- | --- | --- | --- | --- |
| *Decentering and Meditation Methods Interaction Effect and PLEs* | | | | | |
| Term | ß | *SE* | *t* | *p* | 95% CI |
| (Intercept) | 41.94 | 3.85 | 10.88 | < .001*** | [34.34, 49.54] |
| NDM | 1.22 | 0.34 | 3.60 | < .001*** | [0.55, 1.88] |
| CDM | -0.02 | 0.15 | -0.12 | .903 | [-0.32, 0.28] |
| ADM | -0.68 | 0.23 | -2.95 | .004** | [-1.14, -0.23] |
| DEC | -0.26 | 0.12 | -2.15 | .032* | [-0.50, -0.02] |
| Age | -0.14 | 0.03 | -4.67 | < .001*** | [-0.20, -0.08] |
| Sex | 0.80 | 0.82 | 0.98 | .330 | [-0.81, 2.41] |
| NDM × DEC | -0.03 | 0.01 | -2.78 | .006** | [-0.05, -0.01] |
| CDM × DEC | -0.00 | 0.01 | -0.06 | .951 | [-0.01, 0.01] |
| ADM × DEC | 0.02 | 0.01 | 3.13 | .002** | [0.01, 0.04] |
| *Note.* * p < .05, ** p < .01, *** p < .001 | | | | | |
|  | | | | | |

| **S 16** | | | | | |
| --- | --- | --- | --- | --- | --- |
| *Interoceptive Awareness and Meditation Methods Interaction Effect and PLEs in Daily Life* | | | | | |
| Term | ß | *SE* | *t* | *p* | 95% CI |
| (Intercept) | 36.26 | 3.26 | 11.12 | < .001*** | [29.84, 42.68] |
| NDM | 0.95 | 0.30 | 3.16 | .002** | [0.36, 1.53] |
| CDM | 0.12 | 0.13 | 0.94 | .349 | [-0.14, 0.38] |
| ADM | -0.58 | 0.21 | -2.72 | .007** | [-1.01, -0.16] |
| MAIA | -0.03 | 0.03 | -1.00 | .317 | [-0.10, 0.03] |
| Age | -0.13 | 0.03 | -4.64 | < .001*** | [-0.18, -0.07] |
| Sex | 1.06 | 0.75 | 1.41 | .159 | [-0.42, 2.53] |
| NDM × MAIA | -0.01 | 0.00 | -2.32 | .021* | [-0.01, -0.00] |
| CDM × MAIA | -0.00 | 0.00 | -0.98 | .327 | [-0.00, 0.00] |
| ADM × MAIA | 0.01 | 0.00 | 2.78 | .006** | [0.00, 0.01] |
| *Note.* * p < .05, ** p < .01, *** p < .001 | | | | | |
|  | | | | | |

| **S 17** | | | | | |
| --- | --- | --- | --- | --- | --- |
| *Interoception Scores and Meditation in Daily Life* | | | | | |
| Term | ß | *SE* | *t* | *p* | 95% CI |
| (Intercept) | 39.31 | 7.37 | 5.33 | < .001*** | [24.78, 53.83] |
| NDM | 0.27 | 0.25 | 1.06 | .291 | [-0.23, 0.77] |
| CDM | 0.39 | 0.15 | 2.65 | .008** | [0.10, 0.69] |
| ADM | 0.93 | 0.23 | 4.08 | < .001*** | [0.48, 1.37] |
| Age | 0.31 | 0.10 | 3.01 | .003** | [0.11, 0.51] |
| Sex | 5.00 | 2.87 | 1.74 | .083 | [-0.65, 10.65] |
| *Note.* * p < .05, ** p < .01, *** p < .001 | | | | | |
|  | | | | | |

| **S 18** | | | | | |
| --- | --- | --- | --- | --- | --- |
| *NDM Meditation and Reasons to Meditate* | | | | | |
| Term | ß | *SE* | *t* | *p* | 95% CI |
| (Intercept) | 1.20 | 1.12 | 1.08 | .281 | [-0.99, 3.39] |
| SpiritExplore | 0.44 | 0.05 | 8.93 | < .001*** | [0.35, 0.54] |
| Health | 0.03 | 0.03 | 0.98 | .330 | [-0.03, 0.09] |
| Age | 0.04 | 0.02 | 1.79 | .074 | [-0.00, 0.08] |
| Sex2 | -2.13 | 0.55 | -3.90 | < .001*** | [-3.20, -1.06] |
| Sex3 | 5.00 | 3.29 | 1.52 | .129 | [-1.45, 11.46] |

*Note.* * p < .05, ** p < .01, *** p < .001

| **S 19** | | | | | |
| --- | --- | --- | --- | --- | --- |
| *CDM Meditation and Reasons to Meditate* | | | | | |
| Term | ß | *SE* | *t* | *p* | 95% CI |
| (Intercept) | 20.28 | 1.79 | 11.35 | < .001*** | [16.77, 23.79] |
| SpiritExplore | -0.19 | 0.08 | -2.36 | .019* | [-0.34, -0.03] |
| Health | 0.23 | 0.05 | 4.96 | < .001*** | [0.14, 0.33] |
| Age | -0.04 | 0.03 | -1.38 | .168 | [-0.11, 0.02] |
| Sex2 | -2.15 | 0.88 | -2.45 | .015* | [-3.87, -0.43] |
| Sex3 | 8.42 | 5.27 | 1.60 | .110 | [-1.92, 18.76] |

*Note.* * p < .05, ** p < .01, *** p < .001

| **S 20** | | | | | |
| --- | --- | --- | --- | --- | --- |
| *ADM Meditation and Reasons to Meditate* | | | | | |
| Term | ß | *SE* | *t* | *p* | 95% CI |
| (Intercept) | 6.44 | 1.29 | 4.98 | < .001*** | [3.90, 8.98] |
| SpiritExplore | 0.15 | 0.06 | 2.57 | .011* | [0.03, 0.26] |
| Health | 0.19 | 0.03 | 5.70 | < .001*** | [0.13, 0.26] |
| Age | 0.02 | 0.02 | 0.94 | .348 | [-0.02, 0.07] |
| Sex2 | -3.55 | 0.63 | -5.59 | < .001*** | [-4.79, -2.30] |
| Sex3 | 7.18 | 3.81 | 1.88 | .060 | [-0.31, 14.66] |

*Note.* * p < .05, ** p < .01, *** p < .001

**S 21**

*PLEs and Mystical Experiences Model Comparisons for Models with Meditation Categories Only Versus One with Reasons to Meditate Included*

| Model | *F* | *p* |
| --- | --- | --- |
| PLE: Model 1 Vs Model 2 | 11.48 | < .001*** |
| Mystical: Model 1 Vs Model 2 | 8.17 | < .001*** |

*Note.* * p < .05, ** p < .01, *** p < .001. Model 1 contains only the meditation methods (NDM, CDM, ADM) and Age and Sex, and Model 2 also includes the two reasons to meditate factors (SpiritExplore and Health).

| **S 22** | | | | | |
| --- | --- | --- | --- | --- | --- |
| *PLEs Predicted by Meditation and Reasons to Meditate* | | | | | |
| Term | ß | *SE* | *t* | *p* | 95% CI |
| (Intercept) | 38.68 | 1.41 | 27.50 | < .001*** | [35.92, 41.45] |
| SpiritExplore | 0.24 | 0.06 | 3.89 | < .001*** | [0.12, 0.36] |
| Health | 0.03 | 0.03 | 0.82 | .411 | [-0.04, 0.10] |
| NDM | 0.31 | 0.05 | 6.16 | < .001*** | [0.21, 0.42] |
| CDM | -0.07 | 0.03 | -2.12 | .035* | [-0.13, -0.00] |
| ADM | -0.01 | 0.05 | -0.16 | .875 | [-0.10, 0.09] |
| Age | -0.20 | 0.02 | -8.78 | < .001*** | [-0.25, -0.16] |
| Sex2 | -0.46 | 0.64 | -0.72 | .473 | [-1.73, 0.80] |
| Sex3 | -1.12 | 3.78 | -0.30 | .766 | [-8.55, 6.30] |

*Note.* * p < .05, ** p < .01, *** p < .001

**S23**

*Psychotic Like and Mystical Experience Item Correlations*

| CAPE_Pos_Item | Mystical_Item | Correlation |
| --- | --- | --- |
| CAPE_Pos_19 | Mystical#1_26 | 0.37 |
| CAPE_Pos_23 | Mystical#1_24 | 0.36 |
| CAPE_Pos_19 | Mystical#1_25 | 0.33 |
| CAPE_Pos_19 | Mystical#1_5 | 0.29 |
| CAPE_Pos_19 | Mystical#1_24 | 0.29 |
| CAPE_Pos_2 | Mystical#1_3 | 0.27 |
| CAPE_Pos_21 | Mystical#1_25 | 0.27 |
| CAPE_Pos_23 | Mystical#1_27 | 0.27 |
| CAPE_Pos_20 | Mystical#1_12 | 0.27 |
| CAPE_Pos_9 | Mystical#1_25 | 0.27 |
| CAPE_Pos_23 | Mystical#1_25 | 0.27 |
| CAPE_Pos_5 | Mystical#1_24 | 0.27 |
| CAPE_Pos_19 | Mystical#1_7 | 0.26 |
| CAPE_Pos_20 | Mystical#1_5 | 0.26 |
| CAPE_Pos_7 | Mystical#1_30 | 0.26 |
| CAPE_Pos_8 | Mystical#1_3 | 0.25 |
| CAPE_Pos_20 | Mystical#1_13 | 0.25 |
| CAPE_Pos_2 | Mystical#1_5 | 0.25 |
| CAPE_Pos_21 | Mystical#1_23 | 0.24 |
| CAPE_Pos_16 | Mystical#1_5 | 0.24 |
| CAPE_Pos_6 | Mystical#1_27 | 0.24 |
| CAPE_Pos_8 | Mystical#1_10 | 0.24 |
| CAPE_Pos_23 | Mystical#1_7 | 0.24 |
| CAPE_Pos_2 | Mystical#1_7 | 0.24 |
| CAPE_Pos_9 | Mystical#1_23 | 0.24 |
| CAPE_Pos_20 | Mystical#1_1 | 0.23 |
| CAPE_Pos_19 | Mystical#1_27 | 0.23 |
| CAPE_Pos_20 | Mystical#1_10 | 0.23 |
| CAPE_Pos_6 | Mystical#1_3 | 0.23 |
| CAPE_Pos_8 | Mystical#1_1 | 0.23 |

**S24**

*Pre-registered Regression Coefficients After Correction for Violation of Homoscedasticity*

| Pre-registered Hypothesis | P Value |
| --- | --- |
| **Hyp 1a:** |  |
| NDM | <.001*** |
| CDM | <.05* |
| **Hyp 1c:** |  |
| NDM | <.001*** |
| **Hyp 2:** |  |
| Age | <.001*** |
| **Hyp 3:** |  |
| NDM | <.01** |
| Sleep | <.05* |
| DAS | .06 |
| ASI | <0.5* |
| **Hyp 4:** |  |
| Mystical Exp | <.001*** |
|  |  |

*Note.* * p < .05, ** p < .01, *** p < .001

**S25**

**Supplementary Introduction**

Those at risk of psychosis exhibit cognitive biases that are found in people with psychosis. These cognitive biases include aberrant salience, a misattribution of meaning to irrelevant stimuli, heightened attention to threat, and the externalising bias which leads people to misattribute internally generated stimulus to external sources^1^. A genetic predisposition to psychosis has been reported[1], but environmental factors including childhood trauma and immigration status have also been associated with increased risk of psychosis spectrum disorders[2]. Poorer sleep quality has been linked to symptom onset and severity in early psychosis[3], highlighting how environmental factors play a role for those potentially at risk.

In a community sample of teenagers reporting at least one psychotic-like experience, 75% found that experience distressing[4]. Distress from a psychotic episode is reported to stem from both intrapersonal and interpersonal sources[5]. Intrapersonal sources include unwanted internal states, alterations to identity and self, and disruption to goals and behaviours. Interpersonal states included impacts upon relationships, and stigma[5]. Persecutory delusions have been found to be a significant predictor of high anxiety and worry[6]. Indeed, it is reported that heightened anxiety precedes the formation of delusional thinking[7]. Such evidence suggests that PLEs are very likely to cause distress and link to anxiety in both the clinical[6] and non-clinical populations[4], although as we highlighted this might link to ‘appraisals’ of such symptoms and how prevalent paranoid ideation is for the individual[8].

Psychosis has also been reported to lead to ‘personally transformative growth’ for some people[9]. This may happen by taking a purposeful approach to learning from such experiences, and with a ‘mindful’ perspective[9]. Delusions have been suggested to hold personal meaning and help make sense of people's unusual experiences, rather than being nonsensical confabulations, which challenges the traditional view[10]. Religious and spiritual beliefs have been shown to be important aspects of understanding a person's own experience of psychosis, and sometimes alongside a medical diagnosis[11]. This demonstrates perceived distress related to PLEs might vary, and may be linked to attitudes towards such experiences[9] religious and spiritual beliefs[11], persecutory ideation and anxiety[6], but may also lead to personally transformative insights[9].

The discussion about whether mystical experiences might be distressing for some people versus positive for others, requires more attention. Conceptually it can be argued that the alterations of the experience of self, represented by a deep sense of interconnectedness with something greater than one’s self, may share characteristics related to the alteration of the ‘ego’ or self which have been described in relation to PLEs[12]. Parnas and Henriksen[13] in a comparison of PLEs reported by people suffering from schizophrenia with mystical experiences, conclude that a similarity lies in the experience of alterations to the ‘minimal self’ leading to a loss or reduction in self-other distinctions. People seeking a mystical experience via spiritual practices (e.g., meditation) or psychedelics, may attain states that lead to a sense of ‘pure awareness’, but are able to then move back to a normal state of experience. For people suffering from schizophrenia, because they have a core disorder of self, they experience a much more fragmented sense of self that does not recover but persists[13].

It has been suggested that mystical experiences might be more likely to be distressing in people with characteristics common to schizophrenia, including a lower sense of agency over action and ownership of the body[14]. Michalica and Hunt[14] argue that these differences link to less ‘positive presence’. They define it as the qualitative assessment of a sense of embodiment which they measured by asking participants to rate a list of thirty-eight words relating to this concept on how much they represented the ‘fundamental sense of yourself’ including ‘grounded’, ‘drifting’, ‘balanced’ and ‘disconnected’ to name a few. In their study, Michalica and Hunt[14] found a schizophrenic group to score significantly lower on their measure of positive presence versus a control group, although they did not assess if mystical experiences were perceived as more or less positive in the control group. We thus included in our survey a question about how distressing/positive different experiences were for participants, including after the main PLE measure and the mystical experiences measure. However, we did not include this in our write up because of concerns over the potential positive bias of the MEQ and negative bias of the PLE measure (discussed in limitations and discussion of the main sections).

**S26**

**Supplementary Methods**

**Meditation taxonomy correlations**

We ran a Pearson correlation coefficient for all the meditation techniques with PLEs. We then took the top 5 positively correlating techniques and the top 5 negatively correlating techniques and categorised them according to 3 taxonomies. We applied Nash et al’s[15], Dahl et al’s[16] and Matko’s[17] taxonomies to these techniques. We then took the techniques within the categories we defined in our study, and which we used for our main analysis for hypothesis 1a and applied the same approach of categorising according to Mako and Dahl’s taxonomies. The aim was to explore whether there were any patterns in how techniques with either positive or negative correlations to PLEs were categorised according to different taxonomies.

**Multiple comparisons**

With our pre-registered hypotheses, we did not register a correction for multiple comparisons, however we did this post hoc, and all our results remained significant (S1 4). For more details see ‘Controlling for Multiple Comparisons’ in the methods section.

**Pre-registration**

We pre-registered our hypotheses as follows:

1.a) The relationship between lifetime meditation practice and PLEs in 'daily life' will differ depending on the ‘type’ or ‘category’ of meditation techniques most commonly used. Meditation categories will be defined by Nash, Newberg and Awasthi’s (2013)[15] taxonomy, and by Matko’s (2019)[17] classification of techniques based on ‘activation’ and ‘embodiment’.

1.b) The relationship between lifetime meditation practice and PLEs 'during meditation' will differ depending on the ‘type’ or ‘category’ of meditation techniques most commonly used. Meditation categories will be defined as per 1) a).

1.c) The likelihood ratings (that specific meditation techniques will lead to PLEs) will differ depending on the ‘type’ or ‘category’ of meditation techniques most commonly used. Meditation categories will be defined as per 1) a).

2.a) Increased exposure to meditation retreats will positively correlate with PLEs in daily life. Meditation months lifetime experience and frequency of meditation will moderate this relationship, by increasing PLEs with increased months and frequency.

2.b) Increased exposure to meditation retreats will positively correlate with PLEs 'during meditation'. Meditation months lifetime experience and frequency of meditation will moderate this relationship, by increasing PLEs with increased months and frequency.

3) Psychosis proneness will increase the strength of the relationship between meditation and PLEs.

4.a) PLEs will positively correlate with mystical experiences in 'daily life'.

4.b) PLEs will positively correlate with mystical experiences 'during meditation'.

**Statistical analysis and deviations from pre-registration**

**Hypothesis 1 a**

**Model and Variables**

Y = Sum score of PLE positive scale from the CAPE-42 in daily life

X of interest = 3 main regressors representing the lifetime use of techniques grouped into meditation categories according to the Nash, Newberg and Awasthi (2013)[15] taxonomy

X of non-interest = age, sex

Model-comparison was carried out using ANOVA with an ‘empty’ model containing only ‘age’ and ‘sex’ as regressors, and a ‘full model’ with all the regressors defined for the hypothesis. For example for H 1.a) in R we used the formula anova(Hyp1_a_empty_model, Hyp1_a_full_model).

**Deviation from pre-registration**

First, the data collected did not allow us to test hypotheses 1b (similar for 4b below). We had intended to word the question whether participants experienced PLEs during/immediately after meditation as ‘during/immediately after meditation’. However, we accidentally implemented this instead using the question ‘Have you ever associated any of these experiences with the practice of meditation?’’. We did therefore not perform the pre-registered tests and did not count these tests when adjusting for multiple comparisons. Second, we did not repeat the regression analyses using the Matko et al[17] instead of the Nash et al[15] framework because we noted an error in the pre-registered assignment of techniques to four classes (low/high body-focus, etc). However, we applied the Matko et al. classification to the techniques selected for the Nash et al. categories and noticed a strong overlap (table 2). The same applied to all further analyses.

**Hypothesis 1.c.**

We used linear regression models to predict the participants’ likelihood ratings (-5 to 5), i.e. the perceived causality, of the use of different meditation techniques leading to PLEs. Techniques were categorised as described above.

**Model and Variables**

Y = Mean likelihood rating across participants. Specifically, for each participant, their ratings of causality for each technique were summed within categories (i.e. per person, one average causality per technique category).

X of interest = A single categorical regressor indicating 1 of the 3 categories according to either Nash et al.

X of non-interest = age, sex

Model-comparison was carried out using ANOVA with an ‘empty’ model containing only ‘age’ and ‘sex’ as regressors, and a ‘full model’ with all the regressors defined for the hypothesis.

**Hypothesis 2**

We tested the relationship between PLEs and retreat experience, months of lifetime meditation experience and frequency of practice using linear regression.

**Model and Variables**

Y = Sum score of PLE positive scale from the CAPE-42 in daily life

X of interest = Time spent on retreat, months meditating, frequency.

X of non-interest = age, sex

Model-comparison was carried out using ANOVA with an ‘empty’ model containing only ‘age’ and ‘sex’ as regressors, and a ‘full model’ with all the regressors defined for the hypothesis.

**Hypothesis 3**

We tested whether psychosis proneness increased the strength of the relationship between meditation and PLEs using regression analysis. We predicted that psychosis proneness would moderate the relationship between meditation and PLEs.

**Model and Variables**

Y = Sum score of positive scale from CAPE-42

X of interest = ASI and DAS scores, familial psychosis/schizophrenia (Scale of relatedness of family member with diagnosis), childhood trauma rating, sleep quality (SCI scores), migration status (Scale of when moved to current Country of residence) interacted with meditation category scores (as in 1a).

X of non-interest = age, sex

Model-comparison was carried out using ANOVA with a model containing interaction effects of the various psychosis proneness measures upon the relationship between meditation and PLES, and another model without an interaction term.

Model of interest: PLEs ~ + 1 + (NDM + CDM + ADM) * (Nationality + Sleep + DAS + ASI + Trauma + familial psychosis) + Age + Sex.

Control model: PLEs ~ + 1 + (NDM + CDM + ADM) + (Nationality + Sleep + DAS + ASI + Trauma + familial psychosis) + Age + Sex

**Deviation from pre-registration**

We said we wanted to test how psychosis proneness would moderate the relationship between meditation and PLEs. However, we changed our original suggestion for statistical analysis to one that allowed us to specifically scrutinise the influence of psychosis proneness measures upon the relationship of meditation (NDM, CDM, ADM) with PLEs for all participants. Our original suggestion would have required only the use of participants who had a diagnosis of a psychosis spectrum disorder.

**Hypothesis 4 a**

We predicted that PLEs would positively relate to mystical experiences in 'daily life'.

**Model and Variables**

Y = Sum score of positive scale from CAPE-42

X of interest = Mystical Experiences Scale scores

X of non interest = Age, Sex

Statistic of interest: beta coefficient.

**Deviation from pre-registration**

As with hypothesis 1. b, we did not carry out the analysis for PLEs ‘during/immediately after meditation’ for the same reasons described in that section.

**Mystical Experiences Scale**

Mystical experiences have been associated with meditation[18], so the Mystical Experiences Scale[19] was used to capture these experiences, such as feeling connected to all living things, a sense of ineffability and alterations to the experience of time and space. The 30-item revised Mystical Experience Questionnaire (MEQ30) used in this study consists of four subscales and was derived from a forty-three-item version[19]. The MEQ has four subscales including ‘Mystical’, 'Positive Mood’, 'Transcendence of Time and Space’ and ‘Ineffability’. The Mystical subscale (which has items from the internal unity, external unity, noetic quality, and sacredness scales of the MEQ43), relates to the suggestion that ‘mystical’ experiences reported by both religious and non-religious individuals in the literature (e.g. Stace’s 1960 framework) can be characterised predominantly by a deep sense of ‘interconnectedness’ and ‘unity’[20]. This interconnectedness relates both to an internal and external sense of a ‘oneness’ representing ‘God’ or the ‘Universal Self’. The noetic quality aspect relates to a sense that the experience comes from a source of objective truth, noetic meaning of or relating to the intellect. The sacredness aspect relates to the sense the experience is worthy of reverence[20]. The Positive Mood subscale relates to reports of a deep sense of peace and joy accompanying mystical experiences, and the Transcendence of Time and Space subscale relates to experiences of the perception of the boundaries of time and space altering or becoming limitless. The Ineffability subscale relates to the reported quality of mystical experiences being difficult or impossible to put into words or explain to others[20].

Scoring runs from 0-None; not at all to 5-Extreme (more than any other time in my life and stronger than 4), with a highest total of one hundred and fifty with higher scores indicating more self-reported mystical experiences. This measure was designed to assess such experiences whilst using psychedelic substances, which can be seen by the wording of the rating scale. We adapted the instructions at the start of the scale so that it referred to daily experiences, but left the rating scale as it was:

- “Please read each statement and rate how much this has ever applied to you in daily life, and then during/immediately after meditation. The rating scale is as follows:”

The original instructions:

- “Looking back on the entirety of your session, please rate the degree to which at any time during that session you experienced the following phenomena. Answer each question according to your feelings, thoughts, and experiences at the time of the session. In making each of your ratings, use the following scale:”

**Further Control Analyses - regressions**

For each model, we checked the assumptions of linear regression, i.e., linearity (visual inspection of scatter plot of variables of interest), multicollinearity (inspection of correlation tables of regressors, checking for abs(r)>0.5), homoscedasticity (Breusch-Pagan Test) and normality of residuals (Shapiro-Wilk test). We found that all the pre-registered hypotheses violated assumptions of normality and homoscedasticity:

According to Li and Ding[21], the Central Limit Theorem provides that, with sufficiently large sample sizes, the sampling distribution of the mean will approximate a normal distribution, regardless of the shape of the underlying distribution. Given previous work highlighting the robustness of linear regression to violations of normality (other than for small samples, e.g., n<10)[22,23], no corrections were necessary.

**S27**

**Supplementary Figure 1**

Supplementary Figure 1. Significantly correlated items from the CAPE 42+ and the MEQ. Non-significant correlations are not shown, nor are the item self-correlations. Blue hues are negative and red hues are positive, with darker colours depicting stronger correlations.

**References**

1. Bora E, Pantelis C. Theory of mind impairments in first-episode psychosis, individuals at ultra-high risk for psychosis and in first-degree relatives of schizophrenia: systematic review and meta-analysis. Schizophr Res. 2013;144: 31–36. doi:10.1016/j.schres.2012.12.013

2. Dean K, Murray RM. Environmental risk factors for psychosis. Dialogues Clin Neurosci. 2005;7: 69–80. doi:10.31887/DCNS.2005.7.1/kdean

3. Davies G, Haddock G, Yung AR, Mulligan LD, Kyle SD. A systematic review of the nature and correlates of sleep disturbance in early psychosis. Sleep Med Rev. 2017;31: 25–38. doi:10.1016/j.smrv.2016.01.001

4. Kelleher I, Wigman JTW, Harley M, O’Hanlon E, Coughlan H, Rawdon C, et al. Psychotic experiences in the population: Association with functioning and mental distress. Schizophr Res. 2015;165: 9–14. doi:10.1016/j.schres.2015.03.020

5. Griffiths R, Mansell W, Edge D, Tai S. Sources of Distress in First-Episode Psychosis: A Systematic Review and Qualitative Metasynthesis. Qual Health Res. 2019;29: 107–123. doi:10.1177/1049732318790544

6. Startup H, Freeman D, Garety PA. Persecutory delusions and catastrophic worry in psychosis: Developing the understanding of delusion distress and persistence. Behav Res Ther. 2007;45: 523–537. doi:10.1016/j.brat.2006.04.006

7. Freeman D, Garety PA, Kuipers E, Fowler D, Bebbington PE. A cognitive model of persecutory delusions. Br J Clin Psychol. 2002;41: 331–347. doi:10.1348/014466502760387461

8. Baumeister D, Sedgwick O, Howes O, Peters E. Auditory verbal hallucinations and continuum models of psychosis: A systematic review of the healthy voice-hearer literature. Clin Psychol Rev. 2017;51: 125–141. doi:10.1016/j.cpr.2016.10.010

9. Nixon G, Hagen B, Peters T. Psychosis and transformation: A phenomenological inquiry. Int J Ment Health Addict. 2010;8: 527–544. doi:10.1007/s11469-009-9231-3

10. Ritunnano R, Bortolotti L. Do delusions have and give meaning? Phenomenol Cogn Sci. 2022;21: 949–968. doi:10.1007/s11097-021-09764-9

11. Marriott MR, Thompson AR, Cockshutt G, Rowse G. Narrative insight in psychosis: The relationship with spiritual and religious explanatory frameworks. Psychol Psychother Theory Res Pract. 2019;92: 74–90. doi:10.1111/papt.12178

12. Sass LA, Parnas J. Schizophrenia, Consciousness, and the Self. Schizophr Bull. 2003;29: 427–444. doi:10.1093/oxfordjournals.schbul.a007017

13. Parnas J, Henriksen MG. Mysticism and schizophrenia: A phenomenological exploration of the structure of consciousness in the schizophrenia spectrum disorders. Conscious Cogn. 2016;43: 75–88. doi:10.1016/j.concog.2016.05.010

14. Michalica K, Hunt H. Creativity, Schizotypicality, and Mystical Experience: An Empirical Study. Creat Res J. 2013;25: 266–279. doi:10.1080/10400419.2013.813780

15. Nash JD, Newberg AB. An updated classification of meditation methods using principles of taxonomy and systematics. Front Psychol. 2023;13. Available: https://www.frontiersin.org/articles/10.3389/fpsyg.2022.1062535

16. Dahl CJ, Lutz A, Davidson RJ. Reconstructing and deconstructing the self: Cognitive mechanisms in meditation practice. Trends Cogn Sci. 2015;19: 515–523. doi:10.1016/j.tics.2015.07.001

17. Matko K, Sedlmeier P. What Is Meditation? Proposing an Empirically Derived Classification System. Front Psychol. 2019;10. doi:10.3389/fpsyg.2019.02276

18. de Castro JM. Meditation has stronger relationships with mindfulness, kundalini, and mystical experiences than yoga or prayer. Conscious Cogn. 2015;35: 115–127. doi:10.1016/j.concog.2015.04.022

19. Barrett FS, Johnson MW, Griffiths RR. Validation of the revised Mystical Experience Questionnaire in experimental sessions with psilocybin. J Psychopharmacol (Oxf). 2015;29: 1182–1190. doi:10.1177/0269881115609019

20. Maclean KA, Leoutsakos J-MS, Johnson MW, Griffiths RR. Factor Analysis of the Mystical Experience Questionnaire: A Study of Experiences Occasioned by the Hallucinogen Psilocybin. J Sci Study Relig. 2012;51: 721–737. doi:10.1111/j.1468-5906.2012.01685.x

21. Li X, Ding P. General Forms of Finite Population Central Limit Theorems with Applications to Causal Inference. 2018 [cited 15 Apr 2024]. doi:10.6084/m9.figshare.5829645.v1

22. Knief U, Forstmeier W. Violating the normality assumption may be the lesser of two evils. Behav Res Methods. 2021; 2576–2590. doi:10.3758/s13428-021-01587-5

23. Schielzeth H, Dingemanse NJ, Nakagawa S, Westneat DF, Allegue H, Teplitsky C, et al. Robustness of linear mixed-effects models to violations of distributional assumptions. Methods Ecol Evol. 2020;11: 1141–1152. doi:10.1111/2041-210X.13434
