# Supplementary material for: Differential relationship between meditation methods and psychotic-like and mystical experiences
Source: PLoS One. 2024 Dec 5;19(12):e0309357. doi: 10.1371/journal.pone.0309357 (PMC11620446; doi:10.1371/journal.pone.0309357)
Supplement: Attachment — Submitted filename: Reply to reviewers.docx [file pone.0309357.s002.docx]

**To the editor of PLOS One:**

Please find enclosed the newly revised version of our manuscript entitled “Differential relationships between meditation methods and psychotic-like and mystical experiences” that reflects the peer-review comments we have received.

We have taken into account the comments of the two reviewers, whom we thank for their attentive work. We hope that with the changes and overall improvement of the paper, the manuscript might now be considered for publication. We strongly believe that our findings make a substantial contribution to the literature by showing how different meditation techniques can lead to diverse outcomes.

We note that the reviewers’ comments were mostly concerned with providing more discussion in support of our arguments, rather than lack of interest in the conceptual contribution of the work. We now provide the additional evidence requested.

In particular, we have made substantial revisions regarding the presentation of other variables we measured that we consider most relevant, including the ‘reasons to meditate’ data. We have also addressed the question of multiple comparisons, effects sizes, and causality measurements. There is now a substantial and detailed ‘limitations’ section.

You will find in the document submitted with replies to reviewers, detailed responses to their comments. Reviewers’ comments are numbered and in normal font. Responses are in blue, extracts from the original manuscript are in *italic and black* while the revised portions are *in red*.

We would also like to address two comments by the editor, the first highlighting a reviewers’ concerns about the statistical analysis employed. For this, we have included evidence that two of the authors have extensive experience in these methods with published articles to support this claim (outlined in the replies to reviewers). The second comment by the editor relates to a concern about copyright infringement with the use of some images in one of our figures. We have addressed this with a comment submitted on the online system but should also clarify here that we are not aware of any such infringement, and that the figures are created from original illustrations.

**Reviewer #1:**

This is a very interesting but under-researched area of study, and these results have potential clinical implications, so it is welcome that the researchers have decided to conduct this study. Below I describe my main concerns and suggestions.

We thank the reviewer for highlighting that our study is very interesting and has potential impact for clinical applications. We have now amended the manuscript and added discussion sections to provide more supporting evidence for our main conclusions.

1) Causal inference

In the section “Subjective Causality of Meditation Method Use and PLEs” you talk about a criterion for causality citing WHO 2013, “an event that happens concurrently or right after an intervention.” This addresses one of many causal attribution criteria for assessing adverse effects of pharmacological interventions, but it is not really sufficient on its own – please reflect this in your wording. The Varieties of Contemplative Experience paper discusses causal attribution criteria in greater detail (see <https://doi.org/10.1371/journal.pone.0176239> page 28), and subjective judgement of causality is just one of eleven factors used in that study to determine causality.

In discussing causality for this study it is overstretching to talk about a causal relation between meditation method and PLEs. It would be good to consistently use language like “perceived causality” should be used to discuss the finding that participants felt that a method of meditation was causing the experience of a PLE. Additionally, these questions should not be asked of participants who have not had a PLE, because their inclusion in the data skews your results further toward hypothetical perception of a relationship, rather than an empirical one, derived from first-hand experience of those who have had one. You also imply that this relationship is a fact of which participants are subjectively aware in the first paragraph of the discussion. There is not sufficient evidence for this claim.

We thank the reviewer for highlighting the potential issues with our causality assessment, which we initially tried to address using the term “subjective causality” but realise now this might not have been sufficient. We have carefully edited our whole manuscript using the term “perceived causality” and added a limitation section in our results, which we have pasted under the 4th comment of the reviewer.

In order to test the potential issue of including participants who never experienced PLEs in our subjective causality analysis, we also repeated our analysis using only participants who experienced at least 25% of positive symptoms of PLEs (mean CAPE score > 29). Our results did not change. We are now presenting this new analysis in the paper.

***Perceived Causality of Meditation Method Use and PLEs***

*The analyses above establish correlations between the types of meditation and PLEs, but we also wanted to test perceived causality (hypothesis 1c). There are different ways to assess causality (WHO 2013), here we focus on a specific aspect, namely subjective attribution. To assess perceived causality, we asked participants to rate their perception of causality between the different meditation methods and PLEs (Fig. 2D) using a scale running from -5 to 5 (‘Have you found your meditation practice to either make these experiences less likely or more likely to happen?’).*

*We found, indeed, that meditation techniques differed in their perceived causality. First, we related the meditation categories (NDM, CDM, ADM) to subjective ratings of how ‘preventative’ of PLEs the use of techniques within each category were. Doing so, we found that a model with the scores of how preventative each meditation category was differed significantly from one without the categories (F(2) = 18.58, p < .001, S3).*

*Post hoc analysis revealed that all three meditation methods were rated as preventative of PLEs in daily life (S6). However, our results also show that NDM was perceived as the least preventative method compared to the other two (Fig 2D, S6 and 7, NDM vs CDM: t_836_ = -5.48 p < .001; NDM vs ADM: t_836_ = -5.04 p < .001). As a control analysis, to test the perceived causality between meditation categories and PLEs only for those individuals who had experienced PLEs, we repeated the same analysis by only including meditators with at least 25% of PLEs scored as positive (n = 391; Mean CAPE positive score > 29). Our results showed that the perceived causality remained the same; the difference between NDM and CDM was significant (t_1818_ = -5.33, p < .001), as well as NDM and ADM (t_1818_ = -4.86, p < .001) (S8). All meditation methods also remained significantly different from zero (S9).*

We have also added a new section in our Figure 1C to illustrate the distribution of PLEs:

**Figure 1.** A. Meditation methods according to Nash, Newberg and Awasthi’s taxonomy^27^ (NDM= Null Directed Methods, CDM = Cognitive Directed Methods, ADM = Affective Directed Methods). B. Total lifetime use for each meditation method across participants from ratings 0 (Not at all) to 10 (Very often) for each of the five in each category and summed (i.e. minimum is 0 and maximum is 50). A participant could score highly in only one category or in several or in none. C. Distribution of PLE (CAPE positive) scores with a cut-off (red dotted line) for clinical significance . D. Pre-registered analyses: from questionnaires, psychotic-like experiences (PLEs)^15^, and mystical experiences^37^, were extracted to test how they were differentially affected by different types of meditation. E. Spread of overall lifetime meditation practice in years, with the dotted vertical line marking 10+ years experience.

We also added this new analysis to the discussion section:

***Discussion***

*In a pre-registered large online survey (n=613), we tested whether distinct types of meditation methods differentially correlated with PLEs and mystical experiences. We first applied a previous taxonomy based on the goal states of meditation methods^27^ and grouped meditation methods^26^ into Null Directed Methods (NDM), Cognitive Directed Methods (CDM) and Affective Directed Methods (ADM). We found that lifetime use of NDM methods, which aim at achieving an altered state of consciousness devoid of phenomenological content^27^, correlated with PLEs as measured by the CAPE-42 positive symptom scale. Lifetime use of CDM methods showed a negative and statistically significant relationship with PLEs in daily life. We also found that participants were subjectively aware that the different meditation methods have different relationships to adverse experiences, that we term ‘perceived causality’. Indeed, they rated CDM and ADM methods preventative of PLEs, and while NDM was also rated as preventative overall, it was rated as significantly less so than CDM or ADM. This held true for the whole sample (n = 613), as well as for a sample including only those scoring 25% or above on the mean PLE score. Taken together, these findings suggest that the use of meditation methods with a focus on attaining an ‘empty state’ devoid of phenomenological content correlates with an increase in PLEs, while methods aimed at an enhanced cognitive state showed the opposite correlation.*

2) Operationalise Your Terms

a) It would be good to clearly define, for the sake of the study, what definitions you’re using for mystical experiences and PLEs. You do explore the idea that PLEs are the positive aspects of psychosis in a parenthetical statement in your intro, but less discussion is given to what a mystical experience is—perhaps the working definition of both terms is the same and you’re curious how survey participants define them differently. Add a paragraph or two explicitly operationalising your key terms, what working definition you used, and how you measured it (in addition to your later section that describes each of the scales). This is done somewhat in the section called “Differences Between Mystical Experiences in Subjects Distress and Positive Ratings,” but not sufficiently. Are there positive and negative mystical experiences, or have you defined a mystical experience as a positive PLE?

We have now added a new section in the methods to explain our terms more clearly, as:

*Mystical Experiences Scale*

*Mystical experiences have been associated with meditation^35^, so the Mystical Experiences Scale^37^ was used to capture these experiences, such as feeling connected to all living things, a sense of ineffability and alterations to the experience of time and space. The 30-item revised Mystical Experience Questionnaire (MEQ30) used in this study consists of four subscales and was derived from a forty-three-item version^37^. The MEQ has four subscales including ‘Mystical’, 'Positive Mood’, 'Transcendence of Time and Space’ and ‘Ineffability’. The Mystical subscale (which has items from the internal unity, external unity, noetic quality, and sacredness scales of the MEQ43), relates to the suggestion that ‘mystical’ experiences reported by both religious and non-religious individuals in the literature (e.g. Stace’s 1960 framework) can be characterised predominantly by a deep sense of ‘interconnectedness’ and ‘unity’^75^. This interconnectedness relates both to an internal and external sense of a ‘oneness’ representing ‘God’ or the ‘Universal Self’. The noetic quality aspect relates to a sense that the experience comes from a source of objective truth, noetic meaning of or relating to the intellect. The sacredness aspect relates to the sense the experience is worthy of reverence^75^. The Positive Mood subscale relates to reports of a deep sense of peace and joy accompanying mystical experiences, and the Transcendence of Time and Space subscale relates to experiences of the perception of the boundaries of time and space altering or becoming limitless. The Ineffability subscale relates to the reported quality of mystical experiences being difficult or impossible to put into words or explain to others^75^.*

*Scoring runs from 0-None; not at all to 5-Extreme (more than any other time in my life and stronger than 4), with a highest total of one hundred and fifty with higher scores indicating more self-reported mystical experiences. This measure was designed to assess such experiences whilst using psychedelic substances, which can be seen by the wording of the rating scale. We adapted the instructions at the start of the scale so that it referred to daily experiences, but left the rating scale as it was:*

*-	“Please read each statement and rate how much this has ever applied to you in daily life, and then during/immediately after meditation. The rating scale is as follows:”*

*The original instructions:*

*-	“Looking back on the entirety of your session, please rate the degree to which at any time during that session you experienced the following phenomena. Answer each question according to your feelings, thoughts, and experiences at the time of the session. In making each of your ratings, use the following scale:”*

Introduction addition outlining a clearer definition of PLEs and how we measured them:

*Psychotic-like experiences (PLEs) are classified as symptoms that are at a subthreshold for diagnosis for a psychotic episode, with increased prevalence of such experiences signifying an increased risk of psychosis^11^. PLEs are considered part of a continuum of psychosis, and people reporting such experiences do not always report distress or dysfunction^12^, indeed it is estimated that 26.69% of the general population have had at least one PLE^13^ in their lifetime. Yet, PLEs are also a risk factor for transitioning to a psychotic disorder^14^. PLEs can be measured by screening questionnaires such as the CAPE-42^15^ . They consist of positive symptoms presented as hallucinations (visual and auditory), delusions and thought disorder, and negative symptoms including anhedonia, withdrawal from social interaction and depressive symptoms such as rumination^16^. The CAPE-42 has three subscales which are ‘Positive’, ‘Negative’ and ‘Depressive’^17^. We focus here exclusively on the positive subscale, i.e. symptoms related to hallucinations and delusions. Those at risk of psychosis exhibit cognitive biases that are found in people with psychosis. These cognitive biases include aberrant salience, a misattribution of meaning to irrelevant stimuli, heightened attention to threat, and the externalising bias which leads people to misattribute internally generated stimulus to external sources^16^. A genetic predisposition to psychosis has been reported^18^, but environmental factors including childhood trauma and immigration status have also been associated with increased risk of psychosis spectrum disorders^19^. Poorer sleep quality has been linked to symptom onset and severity in early psychosis^20^, highlighting how environmental factors play a role for those potentially at risk.*

b) There is not sufficient treatment of the PLEs at the same time being desirable and a source of insight on the one hand, and on the other, constituting a risk against which certain methods of meditation constitute a protective factor. It would be appropriate to address why some people are seeking PLEs/mystical experiences, and some people may wish to avoid them.

This is a good point regarding why some might see PLEs and Mystical experiences as less distressing/desirable. Our data collection helped answer this question, specifically the ‘reasons to start meditating’ data is relevant here. We have taken two approaches to address this comment, one is to expand on a discussion about the overlap between mystical experiences and PLEs referring to previous literature, and the other is to present data related to subjective distress Vs positive ratings of PLEs and how this links to reasons people started meditating. For the first approach, we will include a discussion related to previous literature in the introduction and discussion sections as outlined below. The reasons to meditate and distress/positive ratings we have included in the results section, and expanded on our factor analysis we ran on the reasons to meditate in the methods section and results. Both are then discussed in the discussion section as highlighted below. We have also addressed a later question about data from other questionnaires not yet included in this article under comment 5) Statistical Analysis.

Introduction addition discussing PLEs, distress and insights, and individual differences:

*In a community sample of teenagers reporting at least one psychotic-like experience, 75% found that experience distressing^38^. Distress from a psychotic episode is reported to stem from both intrapersonal and interpersonal sources^39^. Intrapersonal sources include unwanted internal states, alterations to identity and self, and disruption to goals and behaviours. Interpersonal states included impacts upon relationships, and stigma^39^. Persecutory delusions have been found to be a significant predictor of high anxiety and worry^40^. Indeed, it is reported that heightened anxiety precedes the formation of delusional thinking^41^. Such evidence suggests that PLEs are very likely to cause distress and link to anxiety in both the clinical^40^ and non-clinical populations^38^, although as we highlighted this might link to ‘appraisals’ of such symptoms and how prevalent paranoid ideation is for the individual^42^.*

*Psychosis has also been reported to lead to ‘personally transformative growth’ for some people^43^. This may happen by taking a purposeful approach to learning from such experiences, and with a ‘mindful’ perspective^43^. Delusions have been suggested to hold personal meaning and help make sense of people's unusual experiences, rather than being nonsensical confabulations, which challenges the traditional view^44^. Religious and spiritual beliefs have been shown to be important aspects of understanding a person's own experience of psychosis, and sometimes alongside a medical diagnosis^45^. This demonstrates perceived distress related to PLEs might vary, and may be linked to attitudes towards such experiences^43^ religious and spiritual beliefs^45^, persecutory ideation and anxiety^40^, but may also lead to personally transformative insights^43^.*

Introduction addition discussing mystical experiences, distress and individual differences:

*The discussion about whether mystical experiences might be distressing for some people versus positive for others, requires more attention. Conceptually it can be argued that the alterations of the experience of self, represented by a deep sense of interconnectedness with ‘God’, may share characteristics related to the alteration of the ‘ego’ or self which have been described in relation to PLEs^46^. Parnas and Henriksen^47^ in a comparison of PLEs reported by people suffering from schizophrenia with mystical experiences, conclude that a similarity lies in the experience of alterations to the ‘minimal self’ leading to a loss or reduction in self other distinctions. People seeking a mystical experience via spiritual practices (e.g. meditation) or psychedelics, may attain states that lead to a sense of ‘pure awareness’, but are able to then move back to a normal state of experience. For people suffering from schizophrenia, because they have a core disorder of self, they experience a much more fragmented sense of self that does not recover but persists^47^.*

*It has been suggested that mystical experiences might be more likely to be distressing in people with characteristics common to schizophrenia, including a lower sense of agency over action and ownership of the body^33^. Michalica and Hunt^33^ argue that these differences link to less ‘positive presence’. They define it as the qualitative assessment of a sense of embodiment which they measured by asking participants to rate a list of thirty-eight words relating to this concept on how much they represented the ‘fundamental sense of yourself’ including ‘grounded’, ‘drifting’, ‘balanced’ and ‘disconnected’ to name a few. In their study, Michalica and Hunt^33^ found a schizophrenic group to score significantly lower on their measure of positive presence versus a control group, although they did not assess if mystical experiences were perceived as more or less positive in the control group. We thus included in our survey a question about how distressing/positive different experiences were for participants, including after the main PLE measure and the mystical experiences measure.*

The following is a description of the analysis we ran using the factors from our factor analysis of the reasons people start meditating and the distress ratings of PLEs and Mystical Experiences. This is in the methods section below the title: ‘Exploratory analysis 3: Reasons for meditating, techniques and PLEs’:

*[3] To investigate subjective reports of distress of PLEs, we ran two multiple linear regression analyses with each factor (‘SpiritExplore’ and ‘Health’), the three meditation categories and age and sex as regressors, and distress ratings for PLEs as an outcome for the first, and how positively people rated PLEs for the second. We controlled for the amount of PLEs by adding it to the model (expecting a high correlation with both measures). Our statistic of interest was the beta coefficients for the reason factors.*

We added to the results section to elaborate on our analysis of distress Vs positive experiences of PLEs and Mystical experiences:

*It has been suggested that some meditators are seeking unusual experiences that might lead to insights^29^, which raises the question about if such experiences are judged as positive or distressing. To test this, and to test if there was a difference between mystical experiences versus PLEs, we collected data on subjects' ratings of how distressing and how positive they found these experiences. Two paired sample t-tests revealed mystical experiences were rated as significantly less distressing (t_162_ = -4.52 p = <.001) and as significantly more positive than PLEs in daily life (t_383_ = 9.31 p = <.001, S17). [...] Finally, controlling for the amount of PLEs, we found that people who started meditating for spiritual reasons were more likely to perceive PLEs as positive (t_383_ = 3.61, p = < .001, Fig. 4E, S19), with a non-significant association for ratings of distress (t_383_ = -1.00 p = .32, S18). No relationships were found for the Health factor or for ratings of distress (t_383_ = 1.81 p = .072, S18) or for positivity rating (t_383_ = -0.42 p = .68, S19). For mystical experiences, people who experienced more PLEs perceived mystical experiences as more distressing (t_138_ = 2.95, p <.01, S20), with no difference (or even an opposite trend) to how positively they were perceived.*

We have also added to the methods section a description of our factor analysis of ‘reasons to meditate’ which is outlined below under another comment relevant to this. Below is an addition to the methods section ‘Statistical analysis and deviations from pre-registration’ outlining what the analysis was:

***Exploratory analysis 2: Differences Between Mystical Experiences in Subjects Distress and Positive Ratings***

*We collected data on people’s ratings of how distressing or how positively they rated both PLEs and mystical experiences. We used two paired t-tests to explore if there was significant difference between how people rated mystical experiences Vs PLEs on both dimensions: t.test(PLE Distress Rating, Mystical Distress Rating) and t.test(PLE Positive Rating, Mystical Positive Rating). The statistic of interest was the significance of the p value associated with each test.*

To the discussion section we added discussion about mystical experiences, PLEs, distress and individual differences. We also added discussion about the similarities between the two, starting at the 4th paragraph:

*Meditation is often used by people to create a different perspective about their thoughts, behaviours and emotions, something that can lead to positive changes^29^, and the gaining of insights^56,57^. Our results showed that NDM meditation was related to PLEs and mystical experiences. We also found that PLEs correlate strongly with mystical experiences, supporting previous findings^33^. However, mystical experiences were considered both significantly less distressing and more positive than PLEs by our participants. This supports the idea that unusual experiences, such as those classed as mystical that lead to insights, might be an aim of meditation^29^ and considered beneficial^56,57^. Regarding PLEs, it has been found that people seek clinical support mainly when they exhibit paranoid and persecutory ideation, leading to PLEs being interpreted as threatening^40^. It might be argued that with a more positive appraisal of ‘unusual experiences’, people might be less likely to interpret such experiences as ‘psychotic-like’ and be more biased towards a mystical interpretation. Indeed, PLEs have been found to lead to personally transformative insights for some, which was related to taking a purposeful approach to learning from such experiences and adopting a ‘mindful’ perspective^43^.*

It should be taken into account that the Mystical Experiences Scale used in our study is biased towards collecting positive experiences^58^. It could be argued that mystical experiences are not necessarily positive or negative, and in many cases might be neutral^58^. There is also a suggestion that both PLEs and mystical experiences overlap in terms of their link to altered experiences of ‘self’^47^, and that certain individual traits might influence whether such experiences are considered as positive or negative. For example, amongst people with Schizophrenia such experiences might be more long lasting and distressing^33,47^. Indeed, we found that PLE daily sum was positively associated with distress ratings for mystical experiences, and although not reaching significance this went in the opposite direction for positive ratings.

There is also suggestion that PLEs might be considered negative by some people, but for others do not cause as much distress, and that this might be related to the prevalence of ‘persecutory ideation’^40^. Although we did not measure persecutory ideation, we did collect data about people's motivations to meditate and tested this for any association with how distressing or positively people rated PLEs. Our findings showed for predicting PLEs and mystical experiences, that a model with the reasons to start meditating (SpiritExplore and Health) explained the data better than a model with only the meditation methods (NDM, CDN, ADM). We also found a positive association between self-reported ratings of motivations related to seeking a spiritual experience represented by the SpiritExplore factor and how positively people rated PLEs (S11). No association was found for reasons linked to improving mental health with the Health factor for positive ratings, but a significant positive association was found for how distressing people rated PLEs with the Health factor (S10).

3) Document Layout

a) Please list your hypotheses more clearly at the end of the introduction (as you have done in the pre-registration).

As suggested we added to the end of the introduction an outline of our pre-registered hypotheses:

*We pre-registered our hypotheses as follows (please see Methods for deviation from theses hypotheses):*

*1.a) The relationship between lifetime meditation practice and PLEs in 'daily life' will differ depending on the ‘type’ or ‘category’ of meditation techniques most commonly used. Meditation categories will be defined by Nash, Newberg and Awasthi’s (2013) taxonomy, and by Matko’s (2019) classification of techniques based on ‘activation’ and ‘embodiment’.*

*1.b) The relationship between lifetime meditation practice and PLEs 'during meditation' will differ depending on the ‘type’ or ‘category’ of meditation techniques most commonly used. Meditation categories will be defined as per 1) a).*

*1.c) The likelihood ratings (that specific meditation techniques will lead to PLEs) will differ depending on the ‘type’ or ‘category’ of meditation techniques most commonly used. Meditation categories will be defined as per 1) a).*

*2.a) Increased exposure to meditation retreats will positively correlate with PLEs in daily life. Meditation months lifetime experience and frequency of meditation will moderate this relationship, by increasing PLEs with increased months and frequency.*

*2.b) Increased exposure to meditation retreats will positively correlate with PLEs 'during meditation'. Meditation months lifetime experience and frequency of meditation will moderate this relationship, by increasing PLEs with increased months and frequency.*

*3) Psychosis proneness will increase the strength of the relationship between meditation and PLEs.*

*4.a) PLEs will positively correlate with mystical experiences in 'daily life'.*

*4.b) PLEs will positively correlate with mystical experiences 'during meditation'.*

b) The number of participants that completed the survey is part of the results, in the methods section please explain the study participant selection criteria (eg how did you define “meditator” – was there a minimum required amount of practice?).

We have added to the methods section to clarify how we selected, and defined ‘mediators’: We have also added a plot to figure 1 showing the spread of meditation experience of our sample.

***Participants***

*613 meditators completed the survey up to the end of the meditation habits questions and the main PLE measure (CAPE-42), providing data to test the first hypothesis that different meditation methods will differ in how they relate to PLEs in daily life. We placed a minimum meditation experience of one month, as we aimed to collect data from a sample of people with a range of meditation experience and did not want to limit this to ‘experienced’ or ‘expert’ meditators only. The reason for this relates to our aim to investigate the relationship meditation has to different experiences and personality traits in the general meditator population, to allow the results to be more relevant to a wide range of people.*

*Participants were recruited either via social media channels related to the authors, the University of Plymouth, or the Medito Foundation. The latter also shared a link to the survey within their app (The Medito App). Participants were not paid for taking part in the study, but they could choose a charity to have money donated to on their behalf. 310 participants donated to the Medito Foundation, with the rest selecting a different charity. The recruitment started in February 2022 and stopped in September 2022.*

c) What did you use the motivation to meditate data for? Please clarify.

We have added to the methods section explaining our analysis of the ‘reasons to meditate’ data, and added the results to the results section. We present below the methods section and results section additions, along with an extra figure (Figure 4 presented below).

Methods:

***Factor Analysis for Reasons to Start Meditating***

*We performed exploratory factor analysis with people's responses about how much they endorsed fifteen reasons to start meditating. We implemented factor analysis^84^using the R package “psych”^85^. The aim of factor analysis is to explain correlation between different items of a measure as a function of common underlying factors.*

*The first step was to create a correlation matrix of all fifteen ‘reasons to start meditating’, which is the data the factor analysis seeks to explain. Secondly we used parallel analysis^86^to describe how many factors were appropriate to use. The third step was to perform a factor analysis using generalised least squares to fit the model. The last step implemented “rotating” these factors (by moving the two axes in a 2D coordinate space). This method improves interpretability of the factors. We allowed factors to be correlated (oblique rotation).*

Also we added the following under ‘statistical analysis and deviations from pre-registration’ in the methods section outlining what analysis we ran with the reasons to meditate data:

***Exploratory analysis 3: Reasons for meditating, techniques and PLEs***

*[1] We first tested whether reasons to meditate are differentially related to the techniques people practised. We used a regression for each meditation category as the outcome variable and the two reason factors as the regressors. For example, for NDM: NDM ~ Reason Factor 1 (SpiritExplore) + Reason Factor 2 (Health) + Age + Sex. We reported the beta coefficients for each reason factor as the statistic of interest.*

*[2] We next tested whether there was a relationship between why people started meditating and reports of PLEs and mystical experiences daily, controlling for the techniques they practised. We used a model comparison to test a model with all three meditation categories (NDM, CDM, ADM) and the two reason factors ‘SpiritExplore’ and ‘Health’, with a model with only the meditation categories. Our statistic of interest was the ANOVA result for the model comparison.*

*[3] To investigate subjective reports of distress of PLEs, we ran two multiple linear regression analyses with each factor (‘SpiritExplore’ and ‘Health’), the three meditation categories and age and sex as regressors, and distress ratings for PLEs as an outcome for the first, and how positively people rated PLEs for the second. We controlled for the amount of PLEs by adding it to the model (expecting a high correlation with both measures). Our statistic of interest was the beta coefficients for the reason factors.*

Figure 4 presenting the reasons to meditate results:

*Figure 4. Relationship between reasons to meditate, meditation practices and PLE and mystical experiences. The more people reported starting to meditate for spiritual reasons, the higher the lifetime practice of NDM (A), the higher also the PLEs (C) and mystical (D) experiences during daily life, as well as the positivity ratings for PLEs (E). The more participants reported starting to meditate for health reasons, the higher the lifetime practice of CDM (B). Significance stars relate to p value as follows: * = <.05, ** = <.01, *** <.001.*

Results section presenting the reasons to meditate data:

***Exploratory analysis 3: Reasons for meditating, techniques and PLEs***

*It is plausible that the reasons why people start meditating influence what techniques they use, what meditation experiences they have (PLEs, mystical) and how they perceive them (distressing and/or positively). Therefore, we also asked participants why they started meditating, designing a 15-item questionnaire based on the results of a recent survey study^1^. First, we extracted underlying factors using exploratory factor analysis. Parallel analysis suggested a two-factor solution best fit the data. Factor 1 comprises motivations related to achieving a spiritual experience or exploring unconscious aspects of the mind (Factor ‘SpiritExplore’). Factor 2 contains reasons pertaining to a motivation to achieve improvements in mental health, cognitive abilities, and connection to the body (Factor ‘Health’) (S21).*

*We first tested whether reasons to meditate are differentially related to the techniques people practised. We found that the reason factor SpiritExplore was significantly positively associated with NDM meditation (t_607_ = 8.93, p <.001, Fig. 4A, S22). SpiritExplore was significantly negatively associated with CDM meditation (t_607_ = -2.36, p < .01, S23) whilst reason factor Health was positively associated with CDM technique use (t_607_ = 4.96, p < .001, Fig. 4B, S23). Both factors were positively associated with ADM technique use (Health factor: t_607_ = 5.70, p <.001; SpiritExplore: t_607_ = 2.57, p <.01, S24).*

*We next tested whether there was a relationship between why people started meditating and reports of PLEs and mystical experiences daily, controlling for the techniques they practised. We used a model comparison to test a model with all three meditation categories (NDM, CDM, ADM) and the two reason factors SpiritExplore and Health, with a model with only the meditation categories. We found that for PLEs, the model with the two reason factors was a better fit to the data than the one without (model comparison: F(2) = 11.48, p <0.001, S25). Specifically, SpiritExplore was positively associated with PLEs (t_604_ = 3.89, p < .001, Fig. 4C, S26) while Health was not. For mystical experiences, the model with the reasons factors was likewise a better fit (model comparison: F(2) = 8.17, p = <0.001, S25). SpiritExplore was positively associated with mystical experiences (t_604_ = 4.03, p < .001, Fig. 4D) but Health was not (t_604_ = -1.50, p =.13).*

*Finally, controlling for the amount of PLEs, we found that people who started meditating for spiritual reasons were more likely to perceive PLEs as positive (t_383_ = 3.61, p = < .001, Fig. 4E, S19), with a non-significant association for ratings of distress (t_383_ = -1.00 p = .32, S18). No relationships were found for the Health factor or for ratings of distress (t_383_ = 1.81 p = .072, S18) or for positivity rating (t_383_ = -0.42 p = .68, S19). For mystical experiences, people who experienced more PLEs perceived mystical experiences as more distressing (t_138_ = 2.95, p <.01, S20), with no difference (or even an opposite trend) to how positively they were perceived.*

We added a discussion of the reasons to meditate results in the discussion section (6, 7th paragraphs):

*There is also suggestion that PLEs might be considered negative by some people, but for others do not cause as much distress, and that this might be related to the prevalence of ‘persecutory ideation’^40^. Although we did not measure persecutory ideation, we did collect data about people's motivations to meditate and tested this for any association with how distressing or positively people rated PLEs. Our findings showed for predicting PLEs and mystical experiences, that a model with the reasons to start meditating (SpiritExplore and Health) explained the data better than a model with only the meditation methods (NDM, CDN, ADM). We also found a positive association between self-reported ratings of motivations related to seeking a spiritual experience represented by the SpiritExplore factor and how positively people rated PLEs (S11). No association was found for reasons linked to improving mental health with the Health factor for positive ratings, but a significant positive association was found for how distressing people rated PLEs with the Health factor (S10).*

*We further scrutinised the influence of reasons people meditate by looking at how this influences their choice of meditation technique. We found that SpiritExplore was significantly positively associated with NDM meditation, but significantly negatively associated with CDM meditation. Reason factor Health was positively associated with CDM but not with NDM, and for ADM both factors (Health and SpiritExplore) were significantly positively associated. This suggests people motivated to seek spiritual experiences and explore unconscious aspects of themselves might be more likely to use NDM techniques, which we found linked to PLEs and mystical experiences, with the opposite true for those motivated by improving health and cognitive abilities. Although this fits what we expected, we cannot exclude the fact it is running the other way, which would require further research.*

d) Have your study design precede the description of participants and scales rather than follow them.

We have implemented what was suggested.

e) Also, please clarify what happened to the follow-up questionnaire. You don’t seem to report any results on it.

For our follow up questionnaire we had a technical issue with retrieving the data from the survey platform. Therefore we were unable to test anything for this. We added to the methods section a sentence highlighting this fact:

*This study utilised a repeated measures design employing an online survey. The first version of the survey took approximately one hour to complete with an option to stop halfway through. Using Qualtrics as the survey platform meant that even if participants stopped before the option to finish, their results would be saved once the survey timed out. The second version of the survey was shorter, to help increase the number of people completing it. The questions selected focussed on acquisition of the data to answer the key hypotheses. An option at the end of the survey gave participants a link to another survey where they could leave their email address in order to be contacted for a follow up survey approximately 6 months later. The follow up survey was planned to allow an assessment to be made about changes in key measures in relation to changes in meditation practice such as going on meditation retreat or starting to use a new meditation method. Due to technical issues, a large part of follow-up data was lost; only data from less than 30 participants was available, preventing analysis.*

4) Limitations

a) There is no discussion of the limitations of this study. In your discussion section you talk about the implications and potential application of findings, but there should also be a paragraph that talks about the limitations of causal inference especially in relation to the type of study (cross-sectional survey) and its claims.

You have conducted several statistical analyses, and have not controlled for multiple testing (false discovery rate). Therefore, some of the associations that you found may be spurious, which needs to be acknowledged as a limitation. This is the most important limitation of your work, so please mention it both in the text and in the abstract.

You should also address the degree to which the categories of meditation method are arbitrary and may overlap or be mutually exclusive, as well as any efforts made toward inter-rater reliability in classification of meditator’s self-description of their meditative goals and techniques, as it appears this may have only been done by author TP. Were there any limitations due to your sample population or approach to recruitment?

We added a section to the end of the discussion section addressing the points above:

***Limitations***

*It should be acknowledged that because meditators selected the meditation techniques they practise from the list of fifty descriptions of techniques without any explanation from them about their goals or interpretations of these techniques, individuals may have differed on how they interpreted each technique. For example, one person may select a technique believing it to relate to a specific feature of their tradition, but another may interpret the same technique as one they use in a different tradition. Although this is something to consider, it should be noted that Matko and Sedlmeier’s^26^ list does not preclude the possibility that a description of what someone does using a specific technique within a specific tradition cannot also be the same as a technique with a different name from another tradition. Their aim was to capture ‘what people do when they meditate’ across multiple traditions and techniques and condense this into a smaller list of the most common reports.*

*We assessed correlations between types of meditations and PLEs, as well as perceived causality based on participants' subjective reports of causality. In the future, it could be interesting to obtain objective measures of causality through, for example, a longitudinal design or an intervention study.*

To address the concern of multiple comparisons we added a section at the end of the results section:

***Controlling for Multiple Comparisons***

*To enhance statistical rigour, we pre-registered 4 hypotheses (total of 7 statistical tests - though given some problems with data collection, see section “Statistical analyses and deviations from pre-registration”, this was reduced to 5 tests). While we had not pre-registered multi comparisons across these hypotheses, we nevertheless now include a standard correction procedure^99^ (Benjamini-Hochberg, S3).*

We have added a section to methods that clarifies how meditation techniques were selected for each category (e.g. NDM, CDM, ADM):

***Meditation methods and Categories***

*Participants were shown a list of 50 meditation techniques, based on Matko and Sedlmeier^26^, which is a list derived by asking what meditators do when they meditate. Participants were asked to indicate to what extend they have engaged with each technique with the question: “Please rate all the techniques you selected on how often you use them from '0-Not at all', and '1-A Little' to '10-Very often'. We next extracted from these 50 questions the amount of engagement with each of three categories (NDM, CDM, ADM) according to the Nash, Newberg and Awasthi’s^27^ taxonomy: In step one author TP categorised all of the fifty meditation techniques by placing them in one of the three categories. This was achieved by reading the description of what the meditator does when they use each method and judging whether this indicated an intention to move toward a specific meditative state. The examples given by Nash, Newberg and Awasthi*[*^20^*](https://www.zotero.org/google-docs/?1Hs5x0) *for methods in each category, and in Dahl et al’s^29^ classification system which has considerable theoretical overlap, were referenced with attention paid to specific characteristics of methods in each class. Some techniques are less obviously belonging to one category and therefore overlap with more than one. This initial list was presented to authors EF and JS, who commented and indicated agreement or disagreement for each category, before meeting with TP to discuss. We then produced a second list of meditations placed into the Nash categories that we all agreed upon. In the final step, five methods per category were selected by author TP, which were judged to be most distinctive and easily categorised, and to ensure each category had the same number of methods (table 1). The same process was repeated as for step one, until agreement was reached, and the three categories finalised. We pre-registered this classification. The score for each category (NDM, CDM, ADM) was then computed as the summed endorsement of the five techniques making up each category. To categorize techniques according to Matko et al., we referred to figure 2 in Matko et al.^23^ which shows a 2D matrix of all techniques, along the axes of body orientation and activation. Techniques above each axis’ zero point were labelled as ‘high’, otherwise as ‘low’ (see table 2).*

b) If you have collected your data before registering your analysis plan, unfortunately this does not technically count as pre-registered (even if you have not analysed your data before registration). Please acknowledge this as a limitation.

We have clarified our approach to pre-registration and made it much clearer that data had been collected prior to registration, but not analysed. We agree this is a good point to highlight to ensure misunderstandings don’t occur. We added this to the methods as outlined below:

***Procedure***

*We contacted the Medito foundation who agreed to support distribution of our survey via a link in their meditation app, and via the foundation’s social media channels.* *Data collection started in February 2022, and no analysis on this data occurred before a pre-registration was deposited on the Open Science Forum (OSF) July 2022. We were working within the time constraints of a PhD project, and because we expected an extended time for data collection, we took this approach to allow time for analysis and write up. This was declared. Data was accessible via the survey platform Qualtrics, but we did not look at the data before pre-registration, therefore we adhered to guidelines for pre-registration^72^. Data from a pilot study was used to inform the construction of the scripts for analysis, but this pilot data was not subjected to the full analysis and the data was not included in the final data set.*

c) You state that 613 participants completed at least parts of the survey. How many completed the whole survey, and are there any limitations due to the number that completed it only in part?

We are sorry this was not clear in the method. Our entire survey had twenty blocks of questions lasting more than an hour. Participants were instructed they could stop the survey at the midpoint if they wished to. This was to inform participants that they were nearly finished, with the aim of encouraging them to continue if they wanted to, by allowing time for a breather. The first half of the survey contained all the questions that we needed to address the first two pre-registered hypotheses. If people dropped out before the midpoint we could still use their data as long as they had completed up to the end of a specific questionnaire that was needed.

To address the comment about how many completed the survey, we have added to the descriptive note under Table 2 clarifying how many completed to the end of the survey:

*Note: The ‘coefficient’ column gives correlation coefficients (Pearson’s r) of each meditation technique with PLEs. The table has five techniques for the top positively correlating and top five negatively correlating techniques with PLEs, and a third section showing correlations for the techniques we used for each of meditation categories sorted using the Nash taxonomy. Each technique is then sorted by one of three meditation taxonomies. For Dahl Taxonomy A = Attentional-Family, D = Deconstructive-Family, C = Constructive-Family, FA = Focused-Attention, ND = Non-Dual Oriented Insight, OMo = Open-Monitoring Object-Orientation, R = Relationship-Orientation, P = Perception-Orientation. For Nash Taxonomy NDM = Null Directed Methods, CDM = Cognitive Directed Methods, ADM = Affective Directed Methods. Matko Taxonomy HB = High Body Orientation, LB = Low Body Orientation, HA = High Activation, LA = Low Activation. The colour coding matches up techniques closely linked to each other on ‘body orientation’, ‘activation’ and which cluster they seem most closely related to on Figure 1 of Matko and Sedlmeier’s article What is Meditation? Proposing an Empirically Derived Classification System^26^. If a technique is low in body-oriented focus it will have one of two blue colours, but if high in body orientation it will have one of two red colours. For red ‘high activation’ techniques have the paler red with lower ones a darker red, and for blue techniques low activation have darker blue and higher lighter blue.*

d) You wanted to recruit 1000 participants in order to have a large enough sample size to have participants address 50 different techniques, are there any limitations in the fact that you recruited just over 600? Did you have to make any adjustments in the study design to accommodate this change in sample size?

In our pre-registration we did suggest 1000 as a good number to aim for. However, after 12 months there was a very low frequency of new responses and we decided to stop.

We have addressed the question about recruitment numbers and whether it might affect any analyses done by conducting a post-hoc power analysis for all our pre-registered regressions. The code for this power analysis will be included in the additional R script uploaded to the OSF. We added a paragraph to the end of the methods section explaining what we did.

***Post-hoc Power Analysis***

*Post-hoc power analysis was conducted using the ‘pwr’ package in R^100^. We found that all our pre-registered hypotheses were adequately powered, meaning they all showed a power of > 0.8 (80%), the accepted general standard for power.*

e) Also, the Dahl classification was not pre-registered, please acknowledge this.

We added a sentence in the introduction to clarify that the Dahl et al meditation category approach was not -pre-registered:

*As there is no general agreement about how to best classify meditation techniques^28^ we also classified the meditation techniques according to Matko and Sedlmeier’s^26^ with an emphasis on body orientation (focus on bodily signals) and activation (how active or passive). Finally, we also used the Dahl, Lutz and Davidson^29^ taxonomy which classifies meditation techniques according to their primary cognitive mechanisms including self-enquiry, meta-awareness, cognitive reappraisal and perspective taking^29^. Dahl, Lutz and Davidson’s classification was not pre-registered.*

5) Statistical Analysis

a) It is not clear why you have used the model comparison technique rather than simply looking at the beta coefficients of the predictors of interest in the full multiple regression model. And what statistical test do the t values reported correspond to (ie exactly what are you comparing)? I am not sure you are actually testing what you want to test. Looking at the beta coefficients of your predictors would be a much more direct way (and possibly the only correct way – I am not a statistician) to test your hypotheses.

We agree with the reviewer that statistical tests of regression weights are informative. In fact, we have carried out model comparisons, followed by t-test on regression weights. We have now expanded on this in the methods (see below). We have added a section in the methods under statistical analysis:

***Statistical analysis and deviations from pre-registration***

*The dependent variable from the first and main hypothesis was the positive symptoms scale from the CAPE-42 with two additional items from the LSHS^74^. We checked for collinearity using the variance inflation factor for all the pre-registered hypotheses and they all indicated correlation below a level of concern^87^. Statistical software R^88^ was used and packages required were dplyr^89^, tidyverse^90^, lme4^91^, effects^92^, car^93^, emmeans^94^, sjplot^95^ and ggplot2^96^. Multiple linear regression was used to investigate the relationship between the independent variables and the main PLE measure. We structured our analyses hierarchically^97,98^ i.e. first performed a single statistical test for each pre-registered hypothesis. For most hypotheses, this meant a model comparison of two regression models, one containing the regressors of interest and one not containing them. This was then followed by statistical tests of the individual regression weights. This is conceptually analogous to an ANOVA followed up by post-hoc tests.*

*Hypothesis 1 a*

*In our pre-registration, we predicted that the relationship between lifetime meditation practice and PLEs in 'daily life' (a) or ‘during meditation’ (b) would differ depending on the ‘type’ or ‘category’ of meditation techniques most commonly used. Meditation categories were defined by Nash, Newberg and Awasthi’s (2013) taxonomy, and by Matko’s (2019) classification of techniques based on ‘activation’ and ‘embodiment’.*

*To test (a), we used linear regression models where the sum score of the PLE measure (total score per participant on the CAPE) was predicted by the lifetime use of techniques. These could either be grouped into 3 meditation categories according to the Nash, Newberg and Awasthi (2013) taxonomy or into 4 categories according to Matko and Sedlmeier’s (2019) classification.*

*Model and Variables*

*Y = Sum score of PLE positive scale from the CAPE-42 in daily life*

*X of interest = 3 main regressors representing the lifetime use of techniques grouped into meditation categories according to the Nash, Newberg and Awasthi (2013) taxonomy*

*X of non-interest = age, sex*

*Model-comparison was carried out using ANOVA with an ‘empty’ model containing only ‘age’ and ‘sex’ as regressors, and a ‘full model’ with all the regressors defined for the hypothesis. For example for H 1.a) in R we used the formula anova(Hyp1_a_empty_model, Hyp1_a_full_model).*

*Deviation from pre-registration*

*First, the data collected did not allow us to test hypotheses 1b (similar for 4b below). We had intended to word the question whether participants experienced PLEs during/immediately after meditation as ‘during/immediately after meditation’. However, we accidentally implemented this instead using the question ‘Have you ever associated any of these experiences with the practice of meditation?’’. We did therefore not perform the pre-registered tests and did not count these tests when adjusting for multiple comparisons. Second, we did not repeat the regression analyses using the Matko et al. instead of the Nash et al. framework because we noted an error in the pre-registered assignment of techniques to four classes (low/high body-focus, etc; see S19). However, we applied the Matko et al. classification to the techniques selected for the Nash et al. categories and noticed a strong overlap (table 2). The same applied to all further analyses.*

*Hypothesis 1.c.*

*We used linear regression models to predict the participants’ likelihood ratings (-5 to 5), i.e. the perceived causality, of the use of different meditation techniques leading to PLEs. Techniques were categorised as described above.*

*Model and Variables*

*Y = Mean likelihood rating across participants. Specifically, for each participant, their ratings of causality for each technique were summed within categories (i.e. per person, one average causality per technique category).*

*X of interest = A single categorical regressor indicating 1 of the 3 categories according to either Nash et al.*

*X of non-interest = age, sex*

*Model-comparison was carried out using ANOVA with an ‘empty’ model containing only ‘age’ and ‘sex’ as regressors, and a ‘full model’ with all the regressors defined for the hypothesis.*

*Hypothesis 2*

*We tested the relationship between PLEs and retreat experience, months of lifetime meditation experience and frequency of practice using linear regression.*

*Model and Variables*

*Y = Sum score of PLE positive scale from the CAPE-42 in daily life*

*X of interest = Time spent on retreat, months meditating, frequency.*

*X of non-interest = age, sex*

*Model-comparison was carried out using ANOVA with an ‘empty’ model containing only ‘age’ and ‘sex’ as regressors, and a ‘full model’ with all the regressors defined for the hypothesis.*

*Hypothesis 3*

*We tested whether psychosis proneness increased the strength of the relationship between meditation and PLEs using regression analysis. We predicted that psychosis proneness would moderate the relationship between meditation and PLEs.*

*Model and Variables*

*Y = Sum score of positive scale from CAPE-42*

*X of interest = ASI and DAS scores, familial psychosis/schizophrenia (Scale of relatedness of family member with diagnosis), childhood trauma rating, sleep quality (SCI scores), migration status (Scale of when moved to current Country of residence) interacted with meditation category scores (as in 1a).*

*X of non-interest = age, sex*

*Model-comparison was carried out using ANOVA with a model containing interaction effects of the various psychosis proneness measures upon the relationship between meditation and PLES, and another model without an interaction term.*

*Model of interest: PLEs ~ + 1 + (NDM + CDM + ADM) * (Nationality + Sleep + DAS + ASI + Trauma + familial psychosis) + Age + Sex.*

*Control model: PLEs ~ + 1 + (NDM + CDM + ADM) + (Nationality + Sleep + DAS + ASI + Trauma + familial psychosis) + Age + Sex*

*Deviation from pre-registration*

*We said we wanted to test how psychosis proneness would moderate the relationship between meditation and PLEs. However, we changed our original suggestion for statistical analysis to one that allowed us to specifically scrutinise the influence of psychosis proneness measures upon the relationship of meditation (NDM, CDM, ADM) with PLEs for all participants. Our original suggestion would have required only the use of participants who had a diagnosis of a psychosis spectrum disorder.*

*Hypothesis 4 a*

*We predicted that PLEs would positively relate to mystical experiences in 'daily life'.*

*Model and Variables*

*Y = Sum score of positive scale from CAPE-42*

*X of interest = Mystical Experiences Scale scores*

*X of non interest = Age, Sex*

*Statistic of interest: beta coefficient.*

*Deviation from pre-registration*

*As with hypothesis 1. b, we did not carry out the analysis for PLEs ‘during/immediately after meditation’ for the same reasons described in that section.*

b) You mention that you are not presenting all your results (eg other questionnaires) due to constraints on word count, but PLOS ONE does not have any word count constraints, so please include all your results.

As highlighted above, we have now included our results and write up of the data we collected about the reasons people started meditating. This has included several analyses which we have outlined in the article. Some of the other questionnaires we have not subjected to full analysis, and chosen to focus on those linked to the most coherent and fleshed out hypotheses linked to exploratory analysis (e.g. reasons to meditate, distress and positivity ratings of PLEs and mystical experiences).

**Reviewer #2:**

I appreciate the opportunity to review the manuscript under consideration for publication entitled “Differential relationships between meditation methods and psychotic-like and mystical experiences.” The manuscript is well written and provides novel and important findings for the relationship between meditation practices and psychotic-like experiences. The grouping of meditation practices into different taxonomies is an especially strong characteristic of the study, and allows for a more nuanced picture of the relationship between types of meditation practices and altered state experiences. I am not aware of any prior publications that have conducted such an analysis. Furthermore, the analytic plan is strongly designed and the results are convincing. I believe that this paper provides important information that could be used to identify and monitor risks for adverse effects of meditation. As a result, I recommend this paper for publication considering that the authors address the concerns outlined below:

1) My primary concern is that the manuscript presents an overly simplistic narrative about the relationship between mystical experience and psychotic-like experiences. For example, the discussion in the second to last paragraph of the introduction on the relationship between mystical experience and psychotic-like experiences could benefit from a more critical analysis of the construct of mystical experience (see Taves, 2020). Mystical experience and psychotic-like experiences both represent altered states; however, the mystical experience scale conflates these states with positive affect and positive appraisals, while the measure of psychotic-like experiences that this manuscript uses likely does the opposite. Therefore, it is unsurprising that these two measures of altered states are related to each other (as the results found). Furthermore, differences may be more representative of whether these altered states co-occur with positive or negative affect and/or interpretations (though phenomenology may vary between the scales too). I would like to see more discussion of the similarities and differences (in terms of phenomenology, affect, interpretation) between mystical experience and psychotic-like experience regarding both the general constructs and the specific measures used.

Taves, A. (2020). Mystical and Other Alterations in Sense of Self: An Expanded Framework for Studying Non-ordinary Experiences. Perspectives on Psychological Science 15(3), 669-690. <https://doi.org/10.1177/1745691619895047>.

We have added to the discussion a section discussing the similarities Vs differences between mystical and PLEs. We have also added reference to the positive bias of the MES (Mystical) and the EQ (Decentering), as well as the MAIA2 (interoception).

Mystical:

*It should be taken into account that the Mystical Experiences Scale used in our study is biased towards collecting positive experiences^58^. It could be argued that mystical experiences are not necessarily positive or negative, and in many cases might be neutral^58^. There is also a suggestion that both PLEs and mystical experiences overlap in terms of their link to altered experiences of ‘self’^47^, and that certain individual traits might influence whether such experiences are considered as positive or negative. For example, amongst people with Schizophrenia such experiences might be more long lasting and distressing^33,47^. Indeed, we found that PLE daily sum was positively associated with distress ratings for mystical experiences, and although not reaching significance this went in the opposite direction for positive ratings.*

*There is also suggestion that PLEs might be considered negative by some people, but for others do not cause as much distress, and that this might be related to the prevalence of ‘persecutory ideation’^40^. Although we did not measure persecutory ideation, we did collect data about people's motivations to meditate and tested this for any association with how distressing or positively people rated PLEs. Our findings showed for predicting PLEs and mystical experiences, that a model with the reasons to start meditating (SpiritExplore and Health) explained the data better than a model with only the meditation methods (NDM, CDN, ADM). We also found a positive association between self-reported ratings of motivations related to seeking a spiritual experience represented by the SpiritExplore factor and how positively people rated PLEs (S11). No association was found for reasons linked to improving mental health with the Health factor for positive ratings, but a significant positive association was found for how distressing people rated PLEs with the Health factor (S10).*

2) I would also like to see more description of the interoception and decentering scales used in the manuscript.

This has been fully addressed under comment 3.

Given that these scales were designed to measure adaptive psychological skills, I think it is important to note that they were not designed to be neutral in terms of mental health but instead to measure inherently healthy processes. For example, the MAIA contains the item “I feel that my body is a safe place.” While I do not think that this presents a problem for the present findings, I do think that it is important to note that that these scales are not measuring body awareness and cognitive distancing as unbiased meditation-related processes, but as inherently psychologically healthy skillsets in relation to such processes.

Addition to discussion about ‘decentering’ as a marker of good mental health:

*Reports of psychosis triggered by meditation suggest that psychosis proneness plays a role^9,10^, which is an observation supported by our findings (S8). However, there are also people with no personal or family history of psychosis who have reported to experience a psychotic episode in relation to meditation*[*^9,10^*](https://www.zotero.org/google-docs/?5FsNWl)*. This finding raises the question as to whether other traits in the population might moderate the relationship between the use of certain meditation methods and PLEs, such as those that have been shown to protect against mental ill health. One such trait is called decentering, described as the ability to observe one's own thoughts and feelings from a non-judgemental perspective^59^. This has been suggested to be the main mediating factor between trait mindfulness and its benefits to mental health^60^. Our findings support this idea and suggest that the ability to ‘decenter’ provides a protective factor against PLEs. It should be noted that this trait is considered a marker of good mental health, and therefore is inherently biased towards this as demonstrated by its associations with positive therapeutic outcomes^61^, rather than being a specific outcome of meditation.*

MAIA2 (interoception) and positive associations with body:

*Our findings support this by showing that both the ability to decenter and increased interoceptive awareness moderate the effect of NDM meditation use upon PLEs. This also ties in with evidence that interoceptive awareness is disrupted in schizophrenia and can be characterised as aberrant predictive inference^69^. Such a finding is suggested to provide potential methods for treating psychotic symptoms^69^, such as using mindfulness based interventions which have been shown to have some success in reducing positive psychotic symptoms in schizophrenia^70^. In relation to meditation and psychosis, these findings suggest the practice of CDM and ADM type meditations may be less likely to lead to PLEs, and potentially even act as a protective factor by increasing decentering and interoceptive awareness. The measure we used to test interoceptive awareness leans towards gathering data on positive experiences of the body. Therefore it might be considered an indirect measure of this trait, and similarly it is a self-report measure rather than a physiological test like the ‘Cardioception’^71^ experiment of heartbeat discrimination. Our finding suggests that a more generally positive experience, and increased awareness, of the body moderates the relationship between meditation techniques and PLEs.*

3) For all measures, it would be helpful to include a more thorough description (e.g., subscales, total score) and example items to illustrate what the measure is capturing. I believe that this is important given that the results are dependent on the measures used.

We have added to methods a more thorough description of the measures used:

***Questionnaires Included***

***CAPE-42***

*The community assessment of psychic experiences (CAPE-42) is a 42-item questionnaire designed to measure symptoms of psychosis and was the main psychotic-like experience (PLE) measure. In one study^73^, 3 subscales were found, including positive symptoms (Hallucinations and delusions), negative symptoms (e.g. anhedonia) and depression (e.g. pessimistic thoughts). For this study, questions were clustered into positive symptoms and negative and depressive symptoms, so that additional questions could be included after each. The positive subscale had 3 extra items from the Launay-Slade Hallucination Scale (LSHS)^74^ to ensure visual hallucination experiences were being adequately collected. The CAPE-42 has a 1-4 rating scale from 1-Never to 4-Nearly always to measure frequency. The distress scale was removed, and 1 distress question was added at the end of each subscale from 0-4 to save time in filling in the questionnaire, and because the frequency score is deemed the most important in terms of assessing the prevalence of such experiences.*

***Mystical Experiences Scale***

*Mystical experiences have been associated with meditation^35^, so the Mystical Experiences Scale^37^ was used to capture these experiences, such as feeling connected to all living things, a sense of ineffability and alterations to the experience of time and space. The 30-item revised Mystical Experience Questionnaire (MEQ30) used in this study consists of four subscales and was derived from a forty-three-item version^37^. The MEQ has four subscales including ‘Mystical’, 'Positive Mood’, 'Transcendence of Time and Space’ and ‘Ineffability’. The Mystical subscale (which has items from the internal unity, external unity, noetic quality, and sacredness scales of the MEQ43), relates to the suggestion that ‘mystical’ experiences reported by both religious and non-religious individuals in the literature (e.g. Stace’s 1960 framework) can be characterised predominantly by a deep sense of ‘interconnectedness’ and ‘unity’^75^. This interconnectedness relates both to an internal and external sense of a ‘oneness’ representing ‘God’ or the ‘Universal Self’. The noetic quality aspect relates to a sense that the experience comes from a source of objective truth, noetic meaning of or relating to the intellect. The sacredness aspect relates to the sense the experience is worthy of reverence^75^. The Positive Mood subscale relates to reports of a deep sense of peace and joy accompanying mystical experiences, and the Transcendence of Time and Space subscale relates to experiences of the perception of the boundaries of time and space altering or becoming limitless. The Ineffability subscale relates to the reported quality of mystical experiences being difficult or impossible to put into words or explain to others^75^.*

*Scoring runs from 0-None; not at all to 5-Extreme (more than any other time in my life and stronger than 4), with a highest total of one hundred and fifty with higher scores indicating more self-reported mystical experiences. This measure was designed to assess such experiences whilst using psychedelic substances, which can be seen by the wording of the rating scale. We adapted the instructions at the start of the scale so that it referred to daily experiences, but left the rating scale as it was:*

*-	“Please read each statement and rate how much this has ever applied to you in daily life, and then during/immediately after meditation. The rating scale is as follows:”*

*The original instructions:*

*-	“Looking back on the entirety of your session, please rate the degree to which at any time during that session you experienced the following phenomena. Answer each question according to your feelings, thoughts, and experiences at the time of the session. In making each of your ratings, use the following scale:”*

***Multidimensional Assessment of Interoceptive Awareness***

*To measure interoceptive awareness, a trait found to be influenced by meditation*[*^50^*](https://www.zotero.org/google-docs/?YEi1IU)*, the Multidimensional Assessment of Interoceptive Awareness 2 (MAIA2)*[*^38^*](https://www.zotero.org/google-docs/?AB1MrB) *was presented. The MAIA2 has 8 subscales which are ‘Noticing’, Non-Distracting’, ‘Not-Worrying, ‘Attention Regulation’, ‘Emotional Awareness’, ‘Self-Regulation’, ‘Body-Listening’ and ‘Trusting’^53^. The Non-Distracting subscale has items such as ‘I ignore physical tension or discomfort until they become more severe’. The Not-Worrying scale includes ‘when I feel physical pain, I become upset’ (Reverse scored). We removed the not-worrying and not-distracting scales because we wanted to reduce the overall length of our survey and decided out of the 8 subscales and 36 items that these were the least relevant to Interoception as we understand it. This is based on the fact these scales were aimed at measuring if people distract/worry about uncomfortable bodily sensations, and although relevant, perhaps arguably less relevant than the other six subscales. We acknowledge that without such constraints it is best to include all the subscales.*

*For the other subscales, examples of items included ‘when I am tense, I notice where the tension is located in my body’ (Noticing), ‘I can pay attention to my breath without being distracted by things happening around me’ (Attention Regulation), ‘I notice how my body changes when I am angry’ (Emotional Awareness), ‘when I feel overwhelmed, I can find a calm place inside’ (Self-Regulation), ‘I listen for information from my body about my emotional state’ (Body Listening), ‘I am at home in my body’ (Trusting). The scoring runs from 0-Never to 5-Very Often, with a total (for our survey with the two subscales removed) of one hundred and thirty, with higher scores meaning more of the trait.*

***Experiences Questionnaire***

*To measure ‘decentering', a trait found to be beneficial for mental health, the Experiences Questionnaire was selected^51^. This trait is described as the ability to take a non-judgemental or detached perspective of thoughts and feelings^51^. The EQ has a unifactorial structure with the single factor known as ‘Decentering’. Examples of items in the EQ are ‘I am better able to accept myself as I am’, ‘I am consciously aware of a sense of my body as a whole’, I can take time to respond to difficulties’ and ‘I view things from a wider perspective’^51^ (Fresco et al 2007). Scoring runs from 0-Never to 4-All of the Time, with a potential highest score of forty-four, and higher scores indicating more of the trait.*

***The Somatoform Dissociation Questionnaire-5***

*The Somatoform Dissociation Questionnaire-5 measures dissociative symptoms associated with the body^76^, and was included because PLEs have been linked to a loss of ownership over the body^46,77^. This five-item scale was scored on a 1–5-point scale from ‘1-Not at all’ to ‘5-Quite a bit’. Items included ‘I have pain while urinating’, ‘my body, or part of it, is insensitive to pain’, ‘I see things around me differently than usual (for example as if looking through a tunnel, or seeing merely a part of an object), ‘it is as if my body, or part of it, has disappeared’ and ‘I cannot speak (or only with great effort) or I can only whisper’. The highest total score can be twenty, with higher scores indicating a stronger propensity for this trait.*

***The Sleep Condition Indicator***

*The Sleep Condition Indicator^78^ was used to measure quality of sleep, as sleep deprivation has been linked to PLEs^79^. This eight-item measure includes items such as ‘how long does it take to fall asleep’ (with choices from 0-15 mins to >61 mins), ‘how many nights a week do you have a problem with your sleep?’ and ‘how would you rate your sleep quality?’ from ‘very good’ to ‘very poor’. Items are rated on a five-point scale with varying units of measurement. Higher scores indicate better sleep quality, with a potential highest total score of thirty-two.*

***Hospital Anxiety and Depression Scale***

*The anxiety subscale from the Hospital Anxiety and Depression Scale was used, because increases in anxiety have been associated with an increased chance of having PLEs^54^. Items include ‘I get sudden feelings of panic’, ‘I can sit at ease and feel relaxed’, and ‘worrying thoughts go through my mind’. It is scored from 0-3 from ‘0-not at all’ to ‘3-very definitely and quite badly’ with a total possible score of 21, with higher scores indicating higher anxiety.*

***Aberrant Salience Inventory***

*The Aberrant Salience Inventory measures how much significance and importance is placed on irrelevant stimuli, a characteristic exhibited by those with psychosis^80^, was included. This is a seventeen-item scale scored from 0-5 from ‘o-not at all’ to ‘5-very often’, with the highest total of eighty five, with higher scores indicating more aberrant salience. It consists of one higher order factor, with five lower order factors including ‘Increased Significance’ (F1), ‘Senses sharpening’ (F2), ‘Impending Understanding’ (F3), ‘Heightened Emotionality’ (F4), ‘Heightened Cognition’ (F5). Examples of items include ‘Have you sometimes become interested in people, events, places, or ideas that normally would not make an impression on you?’ (F1), ‘Do your senses sometimes seem sharpened?’ (F2), ‘Do you sometimes feel like you are on the verge of something really big, but you’re not sure what it is?’ (F3), ‘Do you ever feel that your boundaries between inner and outer sensations have been removed?’ (F4) and ‘Do you ever feel like a whole world is opening up to you?’ (F5).*

***Dissociative Absorption Scale***

*The Dissociative Absorption Scale measures how much people tend to focus upon internal stimuli. It is based upon the Absorption and Imaginative Involvement subscale of the Dissociative Experience Scale and is associated with psychopathology^50^. The eight-item scale has a scoring running from 0-10 relating to the percentage of the time that person experiences any of the items. This is presented as ‘0%’ at the start of the scale going up in 10% increments with the final scale point ‘100%’ representing 10 points, with the highest total of eighty, with higher scores indicating more of the trait.*

*Examples of items include ‘Some people have the experience of sometimes remembering a past event so vividly that they feel as if they were reliving that event’, ‘Some people find that when they are watching television or a movie they become so absorbed in the story that they are unaware of other events happening around them’ and ‘Some people find that they sometimes sit staring off into space, thinking of nothing, and are not aware of the passage of time’.*

***The Sense of Agency Scale***

*The Sense of Agency Scale measures to what extent people feel they are initiating and controlling their actions^81^, and has been reported to be diminished in relation to PLEs^77^. This is a seventeen-item scale scored form 1-7 (1-strongly disagree and 7-strongly agree) with the highest total of ninety one. It has a two-factor structure consisting of ‘Sense of Positive Agency’ with item examples including ‘I am in full control of what I do’, and ‘I am the author of my actions’, and ‘Sense of Negative Agency’ including items such as ‘I am just an instrument in the hands of somebody or something else’ and ‘The decision whether and when to act is within my hands’.*

***Kundalini Awakening Scale***

*Six items from the Kundalini Awakening Scale^82^ were selected, to capture experiences reported by meditators in the literature, such as Lindahl et al’s study^83^, that are not distinctly represented by psychosis measures such as the (CAPE-42). This scale was scored from 1-7 (1-strongly disagree and 7-strongly agree) with a highest total of 42, with a higher score indicating more ‘Kundalini Awakening’ experienced by that individual. Item examples include ‘I’ve experienced spontaneous involuntary positioning of the limbs’, ‘I’ve had sensations of tickling, itching or crawling that moved around in a patterned manner’ and ‘I’ve experienced having odd breathing patterns at times’.*

***Additional Scales Used***

*To assess perceived causality, a scale was added to a selected number of psychometrics (CAPE-42, Mystical Experiences, KAS, SoAS) based on reference to validated methods for achieving this for assessing adverse reactions related to pharmacological agents*[*^61^*](https://www.zotero.org/google-docs/?3LH3pG)*. The association between different meditation methods and each set of experiences was assessed by asking participants to give a likelihood rating for the methods they used (‘Have you found your meditation practice to either make these experiences less likely or more likely to happen?’) . The likelihood scale started at -5 indicating a preventative effect (“Very Unlikely”), with 0 as neutral and 5 (“Very Likely”) as causing the experiences to occur. Similarly, for reasons to meditate, a scale from 0-5 was added asking if any of the selected reasons had been linked with the experiences described. To assess the temporal relationship between experiences and meditation, an extra column was added to selected measures asking if these experiences occurred ‘during/immediately after meditation’. This was only done for the mystical experiences, for PLEs it was accidentally phrased as “Have you ever associated any of these experiences* *with the practice of meditation?”. Whether participants considered such experiences as positive or negative was assessed with an extra question. For distress we asked: “Thinking about the questions 1-23 you have just answered, please indicate how distressing you rated these experiences in daily life” with a scale from 0- (“Not Distressed”) to 5- (“Very Distressed”). For positive ratings we asked: “Thinking about the questions 1-23 you have just answered, please indicate how* ***positively*** *you rated these experiences in daily life”, with a scale 0- (Not Positive) to 5- (Very Positive).*

***Meditation methods and Categories***

*Participants were shown a list of 50 meditation techniques, based on Matko and Sedlmeier^26^, which is a list derived by asking what meditators do when they meditate. Participants were asked to indicate to what extend they have engaged with each technique with the question: “Please rate all the techniques you selected on how often you use them from '0-Not at all', and '1-A Little' to '10-Very often'. We next extracted from these 50 questions the amount of engagement with each of three categories (NDM, CDM, ADM) according to the Nash, Newberg and Awasthi’s^27^ taxonomy: In step one author TP categorised all of the fifty meditation techniques by placing them in one of the three categories. This was achieved by reading the description of what the meditator does when they use each method and judging whether this indicated an intention to move toward a specific meditative state. The examples given by Nash, Newberg and Awasthi*[*^20^*](https://www.zotero.org/google-docs/?1Hs5x0) *for methods in each category, and in Dahl et al’s^29^ classification system which has considerable theoretical overlap, were referenced with attention paid to specific characteristics of methods in each class. Some techniques are less obviously belonging to one category and therefore overlap with more than one. This initial list was presented to authors EF and JS, who commented and indicated agreement or disagreement for each category, before meeting with TP to discuss. We then produced a second list of meditations placed into the Nash categories that we all agreed upon. In the final step, five methods per category were selected by author TP, which were judged to be most distinctive and easily categorised, and to ensure each category had the same number of methods (table 1). The same process was repeated as for step one, until agreement was reached, and the three categories finalised. We pre-registered this classification. The score for each category (NDM, CDM, ADM) was then computed as the summed endorsement of the five techniques making up each category. To categorize techniques according to Matko et al., we referred to figure 2 in Matko et al.^23^ which shows a 2D matrix of all techniques, along the axes of body orientation and activation. Techniques above each axis’ zero point were labelled as ‘high’, otherwise as ‘low’ (see table 2).*

4) When reporting regression results, I would like to see some measure of effect size, such as standardised beta.

We ran partial Eta-squared for all the regression results presented in this paper and referenced this once early in the results section under ‘Meditation Method and PLEs in Daily Life’ (and supplementary table S5).

*We tested whether meditation methods differed in their relationship to PLEs (hypothesis 1a). First, we related PLEs in daily life to participants' experience with each meditation category using linear regression (Fig. 1C). Doing so, we found that a model with the use of different meditation methods was a significantly better fit to the data than a model with just age and sex and with no meditation use in (model comparison: F(3) = 29.15, p = <0.001) (Fig. 2A-C, S3). Specifically, our results show that NDM use is significantly and positively related to PLEs (t_606_ = 8.00 p = < .001), CDM use was negatively related to PLEs (t_606_ = -2.30 p = < .05) and there was no significant relationship between ADM and PLEs (S4). Effect sizes were calculated using the partial Eta-squared method, and for NDM this was moderate, CDM small and ADM negligible (see S5 for effect sizes).*

5) The columns in Table 1 (during meditation QUs and Psychosis Proneness QUs) are hard to follow without reading the methods section in detail, which is provided much later in the manuscript. I am still not sure what QU stands for and would like to see this defined in the table note. I would also like to see the three samples described in more detail in the table note, as well as how these differing number of participants relate to the analyses conducted.

We have added extra detail to the note for Table 1. We have also edited the table to remove the ‘during meditation’ sample because we are no longer presenting this. We replaced this with the sample used to test hypothesis 4, and the samples are labelled accordingly. Below is the edited note for this table:

**Table 1** Demographics, Meditation Experience and PLE Score

| Variable | Hypotheses 1.a and c (n = 613) | Hypothesis 3 (n = 155) | | Hypothesis 4 (n = 180) | |
| --- | --- | --- | --- | --- | --- |
| Sex Male | 347 | | 82 | | 95 |
| Sex Female | 262 | | 72 | | 84 |
| Sex Prefer Not to Say | 4 | | 1 | | 1 |
| Mean Age (SE) | 35 (0.55) | | 38.83 (1.2) | | 38.35 |
| Mean Trauma Rating (SE) | 4.07 (0.11) | | 4.22 (0.22) | | 4.29 (0.22) |
| Nationality: Born in Country | 492 | | 119 | | 138 |
| Nationality: Lived Here <10yrs | 48 | | 13 | | 10 |
| Nationality: Lived Here >10yrs or Majority of Life | 73 | | 23 | | 13 |
| *Mean CAPE Pos 20 Items (SE) | 29.55 (0.3) | | 27.48 (0.44) | | 27.52 (0.44) |
| Mean Med Exp Months (SE) | 50.06 (3.1) | | 55.24 (7.5) | | 53.63 (7.28) |
| Participants Who Endorsed NDM | 353 | | 85 | | 101 |
| Participants Who Endorsed CDM | 594 | | 154 | | 178 |
| Participants Who Endorsed ADM | 551 | | 143 | | 167 |

*Note:* Summaries of key demographic variables, meditation experience and positive psychotic symptoms (*Clinical cut-off 50). Participants could complete the whole survey or only parts of the survey. For each hypothesis, the following number of datasets were available: H1a+H1c+H2a – n=613 (Questions about meditation practice and PLE symptoms); H4: n=180 (Included only those who completed the Mystical Experiences questions); H3a: n = 155 (“Psychosis proneness Qus”, i.e. additionally completed questions about risk factors for psychosis). For the purposes of testing the pre-registered hypotheses, we considered a participant to have completed the whole survey to be those who completed it up to the end of the psychosis proneness measures, the last one being the Aberrant Salience Inventory (n = 155). For 'Participants Who Endorsed’ this represents how many picked 1 or more techniques relating to each meditation method. The mean score and standard error for the main psychosis symptom measure (CAPE-42 positive subscale) was 29.55 (0.3), well under 50, the clinical threshold to detect undiagnosed first episode psychosis^48^. 16 participants were above this threshold of 50. ‘Nationality: Born in Country’ is a measure of how recently people have moved to their country of residence.

6) I would also like to see more description of table 2, either in the table note or the results section text, as I find this table hard to follow as well. Please specify exactly what the colors mean and how the rows are organized. It would be helpful to specify that “top positively correlating techniques” refers to correlation with PLEs. I am still somewhat confused by what “techniques used in study” refers to exactly (weren’t all of the techniques used in the study)?

We agree this table needs more elaboration, and we have added extra details to the legend for Table 2 below:

*Note: The ‘coefficient’ column gives correlation coefficients (Pearson’s r) of each meditation technique with PLEs. The table has five techniques for the top positively correlating and top five negatively correlating techniques with PLEs, and a third section showing correlations for the techniques we used for each of meditation categories sorted using the Nash taxonomy. Each technique is then sorted by one of three meditation taxonomies. For Dahl Taxonomy A = Attentional-Family, D = Deconstructive-Family, C = Constructive-Family, FA = Focused-Attention, ND = Non-Dual Oriented Insight, OMo = Open-Monitoring Object-Orientation, R = Relationship-Orientation, P = Perception-Orientation. For Nash Taxonomy NDM = Null Directed Methods, CDM = Cognitive Directed Methods, ADM = Affective Directed Methods. Matko Taxonomy HB = High Body Orientation, LB = Low Body Orientation, HA = High Activation, LA = Low Activation. The colour coding matches up techniques closely linked to each other on ‘body orientation’, ‘activation’ and which cluster they seem most closely related to on Figure 1 of Matko and Sedlmeier’s article What is Meditation? Proposing an Empirically Derived Classification System^26^. If a technique is low in body-oriented focus it will have one of two blue colours, but if high in body orientation it will have one of two red colours. For red ‘high activation’ techniques have the paler red with lower ones a darker red, and for blue techniques low activation have darker blue and higher lighter blue.*

7) Finally, the description of the interaction results is confusing. The sentence “Our results showed interaction between NDM and PLEs in daily life was significantly negative (t207 = -2.78 p = <.01), and positive for ADM and PLEs (t207 = .3.13 p = <.01) (S1 Table 9, Fig. 3D)” seems to be reporting a moderation analysis; however, I think more information is needed to understand this statement. I believe that moderation would involve three variables but I only see mention of two variables in this sentence.

We have changed the wording in the results to clarifying we were looking at moderation. We decided to make extensive changes in this section to make it much clearer, and outline exactly what we did, and what we found:

***Psychosis Proneness, Meditation and PLEs***

*We tested whether the relationship between types of meditation and PLE was moderated by psychosis proneness (hypothesis 3). We captured psychosis proneness as a set of known risk factors for psychosis, namely environmental factors (sleep quality, immigration status and childhood trauma), cognitive characteristics (aberrant salience, dissociative absorption), and familial heritage (family history of psychosis and schizophrenia). To test for moderation, we compared a model containing interactions (i.e. moderating effects) between the three types of meditation and the psychosis proneness factors (S11), with a model without these interactions (S12). We found a model containing the interaction terms was a better fit than one without (F(18) = 2.22, p < .01, S3).*

*Exploratorily, we looked post-hoc at the individual mediating effects. Family history of psychosis and schizophrenia moderated the relationship between NDM meditation and PLEs in a positive direction (t_125_ = 2.58, p < .05, Fig 2F), i.e. in participants with higher family history, there was a stronger relationship between NDM and PLE. Dissociative absorption, a trait previously associated with mental health conditions^50^, showed a moderating effect upon CDM meditation in the negative direction (t_125_ = -3.47, p < .001), and aberrant salience a moderating effect in the positive direction (t_1818_ = 2.63, p < .01) which was also found for nationality (t_125_ = -2.19, p < .05) (how recently people have moved to country of residence) (S11).*

*We wanted to test whether people with a familial predisposition for psychosis were more likely to choose to use meditation methods classified as NDM, to control for the relationship working this way around. To achieve this our third regression analysis had three iterations, with one for each of the meditation methods as the dependent variable and familial history of psychosis spectrum disorders as a regressor along with sex and age. Familial history of psychosis spectrum disorders was not related to any of the meditation methods with NDM (t_151_ = -0.60, p = .55), CDM (t_151_ = -.59, p = .56) and ADM (t_151_ = .22, p = .82).*

To clarify how analysed and presented the moderating effects of ‘decentering’ and ‘interoceptive awareness’ we also made changes to the results section here too:

***Exploratory analysis 1: Decentering, Interoceptive Awareness and PLEs***

*Decentering is a trait suggested to underlie the positive impacts upon mental health of trait mindfulness*[*^36^*](https://www.zotero.org/google-docs/?Od8Lts)*, and we wanted to test if it was preventative of PLEs, and if it moderated the relationship between NDM meditation use and PLEs.*

*We found that the more decentering ability people reported, the lower PLE symptoms (t_207_ = -2.15, p <.05, S14). Decentering also interacted with NDM and ADM. Specifically, decentering moderated the relationship between NDM and PLEs in daily life (t_207_ = -2.78, p <.01, Fig. 3D): the lower the decentering ability, the stronger the link between NDM and PLEs. The interaction between decentering and ADM was also significant (t_207_ = .3.13, p <.01), though note (see above) that there had not been a main effect for ADM in the previous analyses, making the interpretation more difficult. A similar finding was observed with interoceptive awareness, which has been described as the awareness of the global homeostatic state of the body^52^, and measured in our study by the multidimensional assessment of interoceptive awareness 2 (MAIA2)^53^. We found a significant moderating effect in the negative direction for interoceptive awareness and NDM (t_248_ = -2.32, p <.05, Fig 2E) and significant and positive for ADM (t_248_ = 2.78, p <.01) (S15). Further analysis revealed a significant positive relationship between CDM meditation use and interoceptive awareness (t_252_ = 2.65, p <.01) and ADM meditation use (t_252_ = 4.081, p <.001, S16) in daily life.*

**References**

1.	Sedlmeier, P. & Theumer, J. Why Do People Begin to Meditate and Why Do They Continue? *Mindfulness* **11**, 1527–1545 (2020).

2.	Zou, L. *et al.* Effects of Meditative Movements on Major Depressive Disorder: A Systematic Review and Meta-Analysis of Randomized Controlled Trials. *J. Clin. Med.* **7**, 195 (2018).

3.	Goyal, M. *et al.* Meditation Programs for Psychological Stress and Well-being: A Systematic Review and Meta-analysis. *JAMA Intern. Med.* **174**, 357–368 (2014).

4.	Taylor, G. B. *et al.* The Adverse Effects of Meditation-Interventions and Mind–Body Practices: a Systematic Review. *Mindfulness* **13**, 1839–1856 (2022).

5.	Lambert, D., van den Berg, N. H. & Mendrek, A. Adverse effects of meditation: A review of observational, experimental and case studies. *Curr. Psychol.* (2021) doi:10.1007/s12144-021-01503-2.

6.	Farias, M., Maraldi, E., Wallenkampf, K. C. & Lucchetti, G. Adverse events in meditation practices and meditation-based therapies: a systematic review. *Acta Psychiatr. Scand.* **142**, 374–393 (2020).

7.	Goldberg, S. B., Lam, S. U., Britton, W. B. & Davidson, R. J. Prevalence of meditation-related adverse effects in a population-based sample in the United States. *Psychother. Res.* 1–15 (2021) doi:10.1080/10503307.2021.1933646.

8.	Schlosser, M., Sparby, T., Vörös, S., Jones, R. & Marchant, N. L. Unpleasant meditation-related experiences in regular meditators: Prevalence, predictors, and conceptual considerations. *PLOS ONE* **14**, e0216643 (2019).

9.	Charan, D., Sharma, P., Kachhawaha, G., Kaur, G. & Gupta, S. Meditation Practices and the Onset of Psychosis: A Case Series and Analysis of Possible Risk Factors. *Indian J. Psychol. Med.* 02537176211059457 (2022) doi:10.1177/02537176211059457.

10.	Kuijpers, H. J. H., van der Heijden, F. M. M. A., Tuinier, S. & Verhoeven, W. M. A. Meditation-Induced Psychosis. *Psychopathology* **40**, 461–464 (2007).

11.	Kelleher, I. & Cannon, M. Psychotic-like experiences in the general population: characterizing a high-risk group for psychosis. *Psychol. Med.* **41**, 1–6 (2011).

12.	Ermel, J. A., Moran, E. K., Culbreth, A. J. & Barch, D. M. Psychotic like experiences as part of a continuum of psychosis: Associations with effort-based decision-making and reward responsivity. *Schizophr. Res.* **206**, 307–312 (2019).

13.	Bourgin, J. *et al.* Prevalence and correlates of psychotic-like experiences in the general population. *Schizophr. Res.* **215**, 371–377 (2020).

14.	Fusar-Poli, P. *et al.* The psychosis high-risk state: a comprehensive state-of-the-art review. *JAMA Psychiatry* **70**, 107–120 (2013).

15.	Mossaheb, N. *et al.* The Community Assessment of Psychic Experience (CAPE) questionnaire as a screening-instrument in the detection of individuals at ultra-high risk for psychosis. *Schizophr. Res.* **141**, 210–214 (2012).

16.	Livet, A., Navarri, X., Potvin, S. & Conrod, P. Cognitive biases in individuals with psychotic-like experiences: A systematic review and a meta-analysis. *Schizophr. Res.* **222**, 10–22 (2020).

17.	Fonseca-Pedrero, E., Paino, M., Lemos-Giráldez, S. & Muñiz, J. Validación de la Escala para la Evaluación Comunitaria de las Experiencias Psíquicas-42 (CAPE-42) en universitarios y pacientes con psicosis. [Validation of the Community Assessment Psychic Experiences-42 (CAPE-42) in Spanish college students and patients with psychosis.]. *Actas Esp. Psiquiatr.* **40**, 169–176 (2012).

18.	Bora, E. & Pantelis, C. Theory of mind impairments in first-episode psychosis, individuals at ultra-high risk for psychosis and in first-degree relatives of schizophrenia: systematic review and meta-analysis. *Schizophr. Res.* **144**, 31–36 (2013).

19.	Dean, K. & Murray, R. M. Environmental risk factors for psychosis. *Dialogues Clin. Neurosci.* **7**, 69–80 (2005).

20.	Davies, G., Haddock, G., Yung, A. R., Mulligan, L. D. & Kyle, S. D. A systematic review of the nature and correlates of sleep disturbance in early psychosis. *Sleep Med. Rev.* **31**, 25–38 (2017).

21.	Kerr, C., Sacchet, M., Lazar, S., Moore, C. & Jones, S. Mindfulness starts with the body: somatosensory attention and top-down modulation of cortical alpha rhythms in mindfulness meditation. *Front. Hum. Neurosci.* **7**, (2013).

22.	Querstret, D., Morison, L., Dickinson, S., Cropley, M. & John, M. Mindfulness-based stress reduction and mindfulness-based cognitive therapy for psychological health and well-being in nonclinical samples: A systematic review and meta-analysis. *Int. J. Stress Manag.* **27**, 394–411 (2020).

23.	Matko, K., Ott, U. & Sedlmeier, P. What Do Meditators Do When They Meditate? Proposing a Novel Basis for Future Meditation Research. *Mindfulness* **12**, 1791–1811 (2021).

24.	Jachs, B., Garcia, M. C., Canales-Johnson, A. & Bekinschtein, T. A. Drawing the experience dynamics of meditation. 2022.03.04.482237 Preprint at https://doi.org/10.1101/2022.03.04.482237 (2022).

25.	Przyrembel, M. & Singer, T. Experiencing meditation – Evidence for differential effects of three contemplative mental practices in micro-phenomenological interviews. *Conscious. Cogn.* **62**, 82–101 (2018).

26.	Matko, K. & Sedlmeier, P. What Is Meditation? Proposing an Empirically Derived Classification System. *Front. Psychol.* **10**, (2019).

27.	Nash, J. D., Newberg, A. & Awasthi, B. Toward a unifying taxonomy and definition for meditation. *Front. Psychol.* **4**, (2013).

28.	Nash, J. D. & Newberg, A. B. An updated classification of meditation methods using principles of taxonomy and systematics. *Front. Psychol.* **13**, (2023).

29.	Dahl, C. J., Lutz, A. & Davidson, R. J. Reconstructing and deconstructing the self: Cognitive mechanisms in meditation practice. *Trends Cogn. Sci.* **19**, 515–523 (2015).

30.	Sharma, P., Mahapatra, A. & Gupta, R. Meditation-induced psychosis: a narrative review and individual patient data analysis. *Ir. J. Psychol. Med.* 1–7 (2019) doi:10.1017/ipm.2019.47.

31.	Sharma, P., Singh, S., Gnanavel, S. & Kumar, N. Meditation – a two edged sword for psychosis: a case report. *Ir. J. Psychol. Med.* **33**, 247–249 (2016).

32.	Lindahl, J. R. Somatic Energies and Emotional Traumas: A Qualitative Study of Practice-Related Challenges Reported by Vajrayāna Buddhists. *Religions* **8**, 153 (2017).

33.	Michalica, K. & Hunt, H. Creativity, Schizotypicality, and Mystical Experience: An Empirical Study. *Creat. Res. J.* **25**, 266–279 (2013).

34.	Yaden, D., Haidt, J., Hood, R., Vago, D. & Newberg, A. The Varieties of Self-Transcendent Experience. *Rev. Gen. Psychol.* **21**, (2017).

35.	de Castro, J. M. Meditation has stronger relationships with mindfulness, kundalini, and mystical experiences than yoga or prayer. *Conscious. Cogn.* **35**, 115–127 (2015).

36.	Perry, G., Polito, V. & Thompson, W. F. Rhythmic Chanting and Mystical States across Traditions. *Brain Sci.* **11**, 101 (2021).

37.	Barrett, F. S., Johnson, M. W. & Griffiths, R. R. Validation of the revised Mystical Experience Questionnaire in experimental sessions with psilocybin. *J. Psychopharmacol. (Oxf.)* **29**, 1182–1190 (2015).

38.	Kelleher, I. *et al.* Psychotic experiences in the population: Association with functioning and mental distress. *Schizophr. Res.* **165**, 9–14 (2015).

39.	Griffiths, R., Mansell, W., Edge, D. & Tai, S. Sources of Distress in First-Episode Psychosis: A Systematic Review and Qualitative Metasynthesis. *Qual. Health Res.* **29**, 107–123 (2019).

40.	Startup, H., Freeman, D. & Garety, P. A. Persecutory delusions and catastrophic worry in psychosis: Developing the understanding of delusion distress and persistence. *Behav. Res. Ther.* **45**, 523–537 (2007).

41.	Freeman, D., Garety, P. A., Kuipers, E., Fowler, D. & Bebbington, P. E. A cognitive model of persecutory delusions. *Br. J. Clin. Psychol.* **41**, 331–347 (2002).

42.	Baumeister, D., Sedgwick, O., Howes, O. & Peters, E. Auditory verbal hallucinations and continuum models of psychosis: A systematic review of the healthy voice-hearer literature. *Clin. Psychol. Rev.* **51**, 125–141 (2017).

43.	Nixon, G., Hagen, B. & Peters, T. Psychosis and transformation: A phenomenological inquiry. *Int. J. Ment. Health Addict.* **8**, 527–544 (2010).

44.	Ritunnano, R. & Bortolotti, L. Do delusions have and give meaning? *Phenomenol. Cogn. Sci.* **21**, 949–968 (2022).

45.	Marriott, M. R., Thompson, A. R., Cockshutt, G. & Rowse, G. Narrative insight in psychosis: The relationship with spiritual and religious explanatory frameworks. *Psychol. Psychother. Theory Res. Pract.* **92**, 74–90 (2019).

46.	Sass, L. A. & Parnas, J. Schizophrenia, Consciousness, and the Self. *Schizophr. Bull.* **29**, 427–444 (2003).

47.	Parnas, J. & Henriksen, M. G. Mysticism and schizophrenia: A phenomenological exploration of the structure of consciousness in the schizophrenia spectrum disorders. *Conscious. Cogn.* **43**, 75–88 (2016).

48.	Boonstra, T. C. Early detection of psychosis; why should we care? ([s.n.], 2011).

49.	Matko, K., Ott, U. & Sedlmeier, P. What Do Meditators Do When They Meditate? Proposing a Novel Basis for Future Meditation Research. Preprint at https://doi.org/10.31234/osf.io/3xhk8 (2020).

50.	Soffer-Dudek, N., Lassri, D., Soffer-Dudek, N. & Shahar, G. Dissociative absorption: An empirically unique, clinically relevant, dissociative factor. *Conscious. Cogn.* **36**, 338–351 (2015).

51.	Fresco, D. M. *et al.* Initial Psychometric Properties of the Experiences Questionnaire: Validation of a Self-Report Measure of Decentering. *Behav. Ther.* **38**, 234–246 (2007).

52.	Craig, A. D. B. How do you feel--now? The anterior insula and human awareness. *Nat. Rev. Neurosci.* **10**, 59–70 (2009).

53.	Mehling, W. E., Acree, M., Stewart, A., Silas, J. & Jones, A. The Multidimensional Assessment of Interoceptive Awareness, Version 2 (MAIA-2). *PLOS ONE* **13**, e0208034 (2018).

54.	Daniel, C. & Mason, O. J. Predicting Psychotic-Like Experiences during Sensory Deprivation. *BioMed Res. Int.* **2015**, e439379 (2015).

55.	Lindahl, J. R., Kaplan, C. T., Winget, E. M. & Britton, W. B. A phenomenology of meditation-induced light experiences: Traditional buddhist and neurobiological perspectives. *Front. Psychol.* **4**, (2014).

56.	Ko, K., Knight, G., Rucker, J. J. & Cleare, A. J. Psychedelics, Mystical Experience, and Therapeutic Efficacy: A Systematic Review. *Front. Psychiatry* **13**, 917199 (2022).

57.	Carhart-Harris, R. L. *et al.* Psychedelics and the essential importance of context. *J. Psychopharmacol. (Oxf.)* **32**, 725–731 (2018).

58.	Taves, A. Mystical and Other Alterations in Sense of Self: An Expanded Framework for Studying Nonordinary Experiences. *Perspect. Psychol. Sci. J. Assoc. Psychol. Sci.* **15**, 669–690 (2020).

59.	Bernstein, A. *et al.* Decentering and Related Constructs: A Critical Review and Metacognitive Processes Model. *Perspect. Psychol. Sci.* **10**, 599–617 (2015).

60.	Ma, Y. & Siu, A. F. Dispositional mindfulness and mental health in Hong Kong college students: The mediating roles of decentering and self‐acceptance. *Aust. J. Psychol.* **72**, 156–164 (2020).

61.	Bennett, M. P. *et al.* Decentering as a core component in the psychological treatment and prevention of youth anxiety and depression: a narrative review and insight report. *Transl. Psychiatry* **11**, 1–14 (2021).

62.	Seth, A. K. Interoceptive inference, emotion, and the embodied self. *Trends Cogn. Sci.* **17**, 565–573 (2013).

63.	Seth, A. K., Suzuki, K. & Critchley, H. D. An Interoceptive Predictive Coding Model of Conscious Presence. *Front. Psychol.* **2**, (2012).

64.	Fouragnan, E. *et al.* Timing along the cardiac cycle modulates neural signals of reward-based learning. 2022.07.07.498947 Preprint at https://doi.org/10.1101/2022.07.07.498947 (2022).

65.	Fissler, M. *et al.* An Investigation of the Effects of Brief Mindfulness Training on Self-Reported Interoceptive Awareness, the Ability to Decenter, and Their Role in the Reduction of Depressive Symptoms. *Mindfulness* **7**, 1170–1181 (2016).

66.	Fouragnan, E., Queirazza, F., Retzler, C., Mullinger, K. J. & Philiastides, M. G. Spatiotemporal neural characterization of prediction error valence and surprise during reward learning in humans. *Sci. Rep.* **7**, 4762 (2017).

67.	Fouragnan, E., Retzler, C. & Philiastides, M. G. Separate neural representations of prediction error valence and surprise: Evidence from an fMRI meta-analysis. *Hum. Brain Mapp.* **39**, 2887–2906 (2018).

68.	van der Velden, A. M. *et al.* Mindfulness Training Changes Brain Dynamics During Depressive Rumination: A Randomized Controlled Trial. *Biol. Psychiatry* **93**, 233–242 (2023).

69.	Yao, B. & Thakkar, K. Interoception abnormalities in schizophrenia: A review of preliminary evidence and an integration with Bayesian accounts of psychosis. *Neurosci. Biobehav. Rev.* **132**, 757–773 (2022).

70.	Liu, Y.-C., Li, I.-L. & Hsiao, F.-H. Effectiveness of mindfulness-based intervention on psychotic symptoms for patients with schizophrenia: A meta-analysis of randomized controlled trials. *J. Adv. Nurs.* **77**, 2565–2580 (2021).

71.	Legrand, N. *et al.* The heart rate discrimination task: A psychophysical method to estimate the accuracy and precision of interoceptive beliefs. *Biol. Psychol.* **168**, 108239 (2022).

72.	Nosek, B. A., Ebersole, C. R., DeHaven, A. C. & Mellor, D. T. The preregistration revolution. *Proc. Natl. Acad. Sci.* **115**, 2600–2606 (2018).

73.	Brenner, K. *et al.* Validation of the English and French versions of the Community Assessment of Psychic Experiences (CAPE) with a Montreal community sample. *Schizophr. Res.* **95**, 86–95 (2007).

74.	Launay, G. & Slade, P. The measurement of hallucinatory predisposition in male and female prisoners. *Personal. Individ. Differ.* **2**, 221–234 (1981).

75.	Maclean, K. A., Leoutsakos, J.-M. S., Johnson, M. W. & Griffiths, R. R. Factor Analysis of the Mystical Experience Questionnaire: A Study of Experiences Occasioned by the Hallucinogen Psilocybin. *J. Sci. Study Relig.* **51**, 721–737 (2012).

76.	Nijenhuis, E. R. s. The scoring and interpretation of the SDQ-20 and SDQ-5. *Act. Nerv. Super. (Praha)* **52**, (2010).

77.	Tschacher, W., Giersch, A. & Friston, K. Embodiment and Schizophrenia: A Review of Implications and Applications. *Schizophr. Bull.* **43**, 745–753 (2017).

78.	Espie, C. A. *et al.* The Sleep Condition Indicator: a clinical screening tool to evaluate insomnia disorder: *BMJ Open* **4**, e004183 (2014).

79.	Waters, F., Chiu, V., Atkinson, A. & Blom, J. D. Severe Sleep Deprivation Causes Hallucinations and a Gradual Progression Toward Psychosis With Increasing Time Awake. *Front. Psychiatry* **9**, (2018).

80.	Cicero, D. C., Kerns, J. G. & McCarthy, D. M. The Aberrant Salience Inventory: A new measure of psychosis proneness. *Psychol. Assess.* **22**, 688–701 (2010).

81.	Tapal, A., Oren, E., Dar, R. & Eitam, B. The Sense of Agency Scale: A Measure of Consciously Perceived Control over One’s Mind, Body, and the Immediate Environment. *Front. Psychol.* **8**, (2017).

82.	Sanches, L. & Daniels, M. Kundalini and transpersonal development: Development of a Kundalini Awakening Scale and a comparison between groups. **12**, 21 (2008).

83.	Lindahl, J. R., Fisher, N. E., Cooper, D. J., Rosen, R. K. & Britton, W. B. The varieties of contemplative experience: A mixed-methods study of meditation-related challenges in Western Buddhists. *PLOS ONE* **12**, e0176239 (2017).

84.	Yong, A. G. & Pearce, S. A Beginner’s Guide to Factor Analysis: Focusing on Exploratory Factor Analysis. in *Tutorials in Quantitative Methods for Psychology* vol. 9 79–94 (2013).

85.	R: Tools to Accompany the ‘psych’ Package for Psychological Research. https://personality-project.org/r/psych/help/00Index.html.

86.	Hayton, J. C., Allen, D. G. & Scarpello, V. Factor Retention Decisions in Exploratory Factor Analysis: a Tutorial on Parallel Analysis. *Organ. Res. Methods* **7**, 191–205 (2004).

87.	Thompson, C. G., Kim, R. S., Aloe, A. M. & Becker, B. J. Extracting the Variance Inflation Factor and Other Multicollinearity Diagnostics from Typical Regression Results. *Basic Appl. Soc. Psychol.* **39**, 81–90 (2017).

88.	R: The R Project for Statistical Computing. https://www.r-project.org/.

89.	Wickham, H. *et al.* dplyr: A Grammar of Data Manipulation. (2023).

90.	Wickham, H. *et al.* Welcome to the Tidyverse. *J. Open Source Softw.* **4**, 1686 (2019).

91.	Bates, D., Mächler, M., Bolker, B. & Walker, S. Fitting Linear Mixed-Effects Models Using lme4. *J. Stat. Softw.* **67**, 1–48 (2015).

92.	Fox, J. & Hong, J. Effect Displays in R for Multinomial and Proportional-Odds Logit Models: Extensions to the effects Package. *J. Stat. Softw.* **32**, 1–24 (2010).

93.	Fox, J. & Weisberg, S. *An {R} Companion to Applied Regression*. (Sage, 2019).

94.	Lenth, R. V. *et al.* emmeans: Estimated Marginal Means, aka Least-Squares Means. (2023).

95.	Lüdecke, D. *et al.* sjPlot: Data Visualization for Statistics in Social Science. (2023).

96.	Wickham, H. *ggplot2: Elegant Graphics for Data Analysis*. (Springer, 2009). doi:10.1007/978-0-387-98141-3.

97.	Trier, H. *et al.* Emotions and individual differences shape foraging under threat. Preprint at https://doi.org/10.31234/osf.io/v6u3y (2023).

98.	Schlosser, M. *et al.* Measuring Psychological Mechanisms in Meditation Practice: Using a Phenomenologically Grounded Classification System to Develop Theory-Based Composite Scores. *Mindfulness* **13**, (2022).

99.	Chen, S.-Y., Feng, Z. & Yi, X. A general introduction to adjustment for multiple comparisons. *J. Thorac. Dis.* **9**, 1725–1729 (2017).

100.	Champely, S. *et al.* pwr: Basic Functions for Power Analysis. (2020).
