# Supplementary material for: Differential relationship between meditation methods and psychotic-like and mystical experiences
Source: PLoS One. 2024 Dec 5;19(12):e0309357. doi: 10.1371/journal.pone.0309357 (PMC11620446; doi:10.1371/journal.pone.0309357)
Supplement: Attachment — Submitted filename: Responses to Reviewer Comments.docx [file pone.0309357.s003.docx]

Reviewer #1:

 Thanks again for inviting me to comment on this interesting work. This new version represents significant progress. The authors have worked hard adding important information in a number of places. I am afraid, however, that the manuscript needs further work, as detailed below.

• The paper is very hard to parse, it needs more attention in order to avoid confusion. For example, some methods are explained in the introduction and in the results. Also, results are presented without introducing them in methods: Table 2 presents pearson correlations not introduced in the methods or linked with anything else.

We agreed with the reviewer that the paper was very dense after the revisions. We have tried to clarify the text as much as possible to avoid duplicates. We have also created a supplementary methods section where Table 2 Pearson’s correlation are explained:

**Meditation Taxonomy Corelations**

We ran a Pearson correlation coefficient for all the meditation techniques with PLEs. We then took the top 5 positively correlating techniques and the top 5 negatively correlating techniques and categorised them according to 3 taxonomies. We applied Nash et al’s^23^, Dahl et al’s^24^ and Matko’s^25^ taxonomies to these techniques. We then took the techniques within the categories we defined in our study, and which we used for our main analysis for hypothesis 1a and applied the same approach of categorising according to Mako and Dahl’s taxonomies. The aim was to explore whether there were any patterns in how techniques with either positive or negative correlations to PLEs were categorised according to different taxonomies.

• Plos One papers have the methods before the results, please move them.

We have now done so.

• Regression weights and coefficients are not the same, please use word coefficients consistently.

We agree it is best to use a consistent term throughout and have replaced weights with coefficients throughout.

• Please clarify in the methods that the t tests are on regression coefficients. As far as I understand, what is usually reported in regressions is the standardised beta coefficient and the p value, not the t value. And why are effect sizes calculated using partial eta squared and not simply interpreting the regression coefficients? I suggest you report the beta coefficients instead of the t values, as they are more interpretable. it is good practice to report 95% confidence intervals too.

We chose eta-squared over standardized beta coefficients as this has a more intuitive interpretation and there are standard tables to characterize effect sizes as ‘small’, ‘medium’ or ‘large’. Standardized beta coefficients (i.e. the regression coefficients obtained when regressors are standardized [i.e. subtracting the mean, dividing by standard deviation]) can range from minus to plus infinity, thus are less intuitive. We include beta coefficients in the regression output tables in the sup.

• Have the authors tested whether regression models were meeting statistical assumptions (eg distribution of residuals, etc)? There is no mention of this important aspect that can invalidate results if assumptions are not met.

We have now addressed this concern with a section in the supplements, and a results table of a new analysis controlling for heteroscedasticity as S24 in the supplements:

**Further Control Analyses - regressions**

For each model, we checked the assumptions of linear regression, i.e., linearity (visual inspection of scatter plot of variables of interest), multicollinearity (inspection of correlation tables of regressors, checking for abs(r)>0.5), homoscedasticity (Breusch-Pagan Test) and normality of residuals (Shapiro-Wilk test). We found that all the pre-registered hypotheses violated assumptions of normality and homoscedasticity. As mentioned in the main text, there were no issues with multicollinearity.

According to Li and Ding^42^, the Central Limit Theorem provides that, with sufficiently large sample sizes, the sampling distribution of the mean will approximate a normal distribution, regardless of the shape of the underlying distribution. Previous work has highlighted the robustness of linear regression to violations of normality (other than for small samples, e.g., n<10)^43,44^. Despite this fact, we decided to add a control test to account for violations of homoscedasticity. To compute the significance of the regression coefficients we used heteroscedasticity-consistent (HC) standard errors implemented in the sandwich package in R^45^. We find that all regression coefficients remain significant in our pre-registered hypotheses with one exception for hypothesis 3 (psychosis proneness measures) with the dissociative absorption (DAS) score. This changed from a significance of p <.05 to .06 (S24).

• Reasons for meditating seem to have been classified into two factors, but there is no explanation of what these factors are (only variable names in the graphs).

We apologise for the lack of clarity. This was in the methods, on section titled “Factor Analysis for Reasons to Start Meditating”(page 6). We hope that with the restructuring (methods first) this is now easier to find. There is a table in the supplements with these reasons placed into relevant factors, that we have referred to in the text (S3).

• Regarding making causal claims about meditation type and PLEs: The section on hypothesis 1c seems clear with regards to perceived causality, but in the Discussion (on page 3) authors argue they have evidence for causality, "We also found that participants were subjectively aware that the different meditation methods have different relationships to adverse experiences, that we term ‘perceived causality’." this should rather be stated, "Although cross-sectional survey data can't prove a causal relationship between meditation type and PLEs, we have found that meditators perceive that different types of meditation may be more likely to elicit PLEs than others."

This has been changed as suggested.

• They have a much better discussion of the potential connections and differences between mystical experiences and PLEs.

We thank the reviewer for their positive comment.

• I'd encourage them to use one word like "perceive" just in context of subjective causal attribution and different word like "appraise" in terms of subjective valence (pos/neg), otherwise. For instance this sentence on the top of page 10, "For mystical experiences, people who experienced more PLEs perceived mystical experiences as more distressing (t138 = 2.95, p <.01, S20), with no difference (or even an opposite trend) to how positively they were perceived."

This has been changed as suggested. The references to distress and PLEs Vs mystical have been removed in relation to a comment by the other reviewer.

• The limitations section needs much more thought. Important ones that are not mentioned, are that the cross-sectional nature does not allow to understand the temporal sequence of factors, and that these relationships can all be confounded by unmeasured factors. Both have important implications that need to be reflected upon and discussed.

We have added two important sections in the limitations section as suggested (Page 22).

Reviewer #2:

The authors have extensively reworked this manuscript, and these changes have led to a significant improvement. However, a number of problems remain which I would like to see addressed before recommending this manuscript for publication.

1. General comments:
   a. While some of the additional information provided in this version of the manuscript is important, some of the sections have become quite wordy and excessively long. For example, the scale descriptions in the methods section are an important addition, but need to be further edited to be more concise and parsimonious. Similarly, the analysis section of the methods feels excessively long and in need of further editing. I would like to see this section written in full sentences and paragraph form.

We have a more succinct description of measures in the main article (Page 6), with a fuller more detailed description in the supplementary methods section (Questionnaires Included). We have edited the analysis section of the main article (Page 8 up to and including Page 11) and included a more detailed version in the supplementary methods section (Statistical analysis and deviations from pre-registration). The section in the main article is now cleared titled and more succinct, written in full sentences.

Here is an example of how we have now formatted it in the main text:

**Meditation Method and PLEs (hypothesis 1a)**

We used multiple linear regression to investigate the relationship between types of meditation (independent variables) and the main daily-life PLE measure (positive scale from CAPE-42 and 3 items about visual hallucination from LSHS^36^). The dependent variable was the sum score of the PLE measure (total score per participant on the CAPE). The independent variables were meditation categories defined according to Nash et al^22^ i.e. 3 categories . Pre-registered hypothesis 1b, i.e. the same test for PLEs during meditation could not be tested due to an error in phrasing the question (see methods section in supplements).

PLEs ~ NDM Meditation Use + CDM Meditation Use + ADM Meditation Use + Age + Sex

b. The manuscript could benefit from additional proofreading, as there are awkward sentences, typos, and grammatical errors throughout.

Thank you for your careful read, we have now reviewed our entire paper for typos and grammatical issues.

c. The header structure is at times unclear and confusing. For example, in the methods section, it appears (but I’m not sure) that “Questionnaires Included” is a separate section after “Materials and Measures.” I would recommend using a more standard header structure and providing some additional subheaders in the introduction and discussion. For example, the transition from the second to third paragraph of the introduction feels very abrupt and could either benefit from a header or further editing.

We have now substantially rewritten the introduction (Page 3 up to and including Page 4) which is now shorter and edited the methods section (Page 5 up to and including Page 11). We believe that it reads a lot better now.

d. The new sections of the introduction on the valence of psychotic or mystical experiences also seem unnecessarily long for this point in the introduction (see additional comments below about this novel section). As a general point, I think the introduction needs additional framing to contextualize some of the results that are not introduced at all in the introduction, while this novel section is important but currently overrepresented in the introduction.

We agree with the reviewer. Please see comment above as this is relevant for this new one. We have removed a lot of this discussion relating to valence of PLEs and mystical experiences. We also stated how we are specifically measuring these experiences:

Here we measure mystical experiences using the Mystical Experiences Questionnaire (MEQ) and psychotic-like experiences with the Community Assessment of Psychic Experiences-42 (CAPE) positive subscale with three extra items from the Launay-Slade Hallucination Index (LSHI)^36^.

2. Theoretical Issues:
   a. There are theoretical problems in the novel section of the introduction about the valence of psychotic and mystical experiences. Unfortunately, the authors seem to have misunderstood my previous point about the overlap between mystical and psychotic-like experiences. Page 3 of the introduction discusses the incidence of distress vs. “personally transformative growth” as a result of psychosis. However, this conflates the general construct of “psychosis” with the specific CAPE-42 items about hallucinations and delusions that this study uses as its measure of PLEs. “Psychosis” is already a pathologizing term, and as a clinical diagnostic category, it is defined in terms of functional impairment or distress. Instead, I would like to know whether the CAPE-42 items used in the current study are worded in a way that assumes/implies they are negative/pathological or whether they are phenomenologically neutral. This is important, as visions and magical thinking (another way of phrasing hallucinations and delusions) are valued in many spiritual traditions. If the items are neutrally worded, it is therefore unclear whether they are measuring something innately pathological or not.

We agree this is a complex issue, and it may be that people may frame or interpret the same type of experience in different ways, and that levels of distress associated with such experience may vary too. We have tried to discuss this point, including a new analysis looking at the wording of the items in the CAPE-42+ and the MEQ and the correlation of these items.

In short, we believe that even though there can be more “negatively” phrased items in the CAPE-42 and more “positively” phrased items in the MEQ, there remains an item-by-item relationship between the two questionnaires which indicate that generally, regardless of the valence of the items used, people that are more likely to experience PLEs and also the ones that are more likely to experience mystical experiences, which was our original point. We, however, take on board the suggestion and included the additional analyses in results and discussion. We have also streamlined our introduction and removed reference to personally transformative growth.

Another point is that we think the distinction between PLEs and psychosis is important, and have purposefully not used the word psychosis but only PLEs. It is true that PLEs are often reported as distressing, and we thank the reviewer for raising this important point regarding ‘pathology’. We previously included discussion on this point and have now attempted to make this much clearer. This also ties in with a discussion we have also included about negative versus positive biases in terms of specific questionnaires (CAEP-42 and MEQ), and our new analysis.

We have included a new analysis an outlined this in the methods section:

**Exploratory analysis 3: Correlations of PLE and mystical measure items (CAPE-42 +3 LSH and the MEQ), and theoretical overlap**

We used all the CAPE-42+ items and the MEQ items to create a correlation matrix (Pearson’s r) to explore the links between individual items of our PLE mystical experiences measures.

Please see results section titled (Page 19 and 20):

**Exploratory analysis 3: Correlations of Questionnaire Items from the CAPE-42+ and the MEQ**

Our discussion section now includes a discussion of our new analysis in the 4^th^ paragraph.

b. The next paragraph about mystical experience in the introduction has the same theoretical problems. Given that this study uses the MEQ30 to measure mystical experiences, mystical experiences should be understood as whatever is being measured by these items (which notably do not include the word God). Therefore, I would like to see the discussion of mystical experiences less tied to an abstract notion and more related to what is specifically being measured in this study. More importantly, the MEQ includes positive affect in its definition, which makes a discussion of whether mystical experiences are positive for some people or distressing for others nonsensical when based on this measure and definition, given that the construct is *defined* as being innately positive. I would encourage a critique of the way that mystical experiences have been defined, perhaps in the discussion or limitations, but given that this definition is already being used, it does not make theoretical sense to discuss its valence. Finally, please replace the Parnas & Hendriksen citation with a more up-to-date citation on the sense-of—self-related similarities between mystical and pathological experiences or leave out this citation entirely, as current scholarship on the topic has advanced considerably since this was published.

We have included the aforementioned new analysis of the item-by-item relationship of the MEQ and CAPE as discussed. This includes discussion of the valence of each questionnaire. The analysis of distress ratings of MEQ and CAPE has been removed. The citation mentioned is removed from the main text too.

c. Here is what I would recommend in response to these issues: introduce the MEQ and CAPE-42 as being innately biased toward positive or negatively valenced experience (if that is also the case for the CAPE-42) and comment on how they share some common phenomenology (if they do, at the item level). Then, throughout the rest of the paper, frame PLEs and mystical experience as related but each biased toward pathological/negative appraisals or spiritual/positive appraisals.  Finally, the “exploratory analysis 2: differences between mystical experiences in subjects distress and positive ratings” should be removed, as it is redundant to ask about the valence of mystical experiences when the measure already includes positive affect.

We thank the reviewer for the suggestion. Our response to the previous two comments links to this. We have discussed potential phenomenological overlaps, and differences.

The section on the discussion that covers these topics also needs to be edited based on the issues identified above. Please discuss what is actually being measured in this study rather than assuming that it refers to broader abstract concepts. For example:

i.The sentence “It could be argued that mystical experiences are not necessarily positive or negative and, in many cases, might be neutral” doesn’t make sense when mystical experiences are *defined* as involving positive affect, though authors have pointed out that this definition is problematic (see Taves, 2020).

ii.“PLEs and mystical experiences overlap in terms of their link to altered experiences of self” – please specify if this is the case, and if so which experiences of self, for the two specific measures used in this study.

iii.Likewise, please link the discussion of interoception more closely to the specific items/subscales used to measure interoception. For example, feeling safe in one’s body might not be the same thing as the felt sense of presence in one’s body (see self review article: Britton et al., 2021). There are many forms of interoception and it is problematic to assume that the measure that this study uses for interoception corresponds to theoretically different conceptualizations of interoception.

We thank the reviewer for their detailed analysis and examples of specific areas of concern, and suggestions to address them. We have made substantial additions to the discussion (and results) to clarify the specific overlaps (or not) that our chosen measures seem to have regarding wording and correlation of items and think this is useful addition. Some of the discussion around the concept of a mystical experience and how it relates to PLEs has been kept but edited significantly. We have clearly outlined the considerations regarding how we measured mystical experiences and linked it to this wider discussion (also included in limitations).

We hope the additional analysis (Results Pages 19 and 20) and discussion (Discussing Page 21 and 22) gives a more specific identification of overlap of our chosen measures, and addresses some of these concerns.

3. Issues with framing the results:
   a. The hypothesis section of the introduction feels quite lengthy and many of the hypotheses were not framed or supported in the introduction until that point. I am confused by why there is a paragraph describing hypotheses and then a long list of many of the same hypotheses below. Maybe the longer list could be moved to supplemental materials. I also think 1a, 1b, and 1c could be condensed into a single paragraph perhaps.

The method section has been substantially shortened and rewritten. We have kept all details of the analyses done including equations in the supplementary methods.

b. I am confused about why the methods section is below the results and discussion. I think it would be easier to follow the results if I understood the methods first, especially the measures section (with example items to illustrate exactly what is being measured).

This has been changed.

c. In the results section, I think it would read better if much of the information in the table/figure captions was moved to the body text. The table/figure captions are very long and hard to follow and the paragraphs do little to introduce the tables/figures.

We have done our best to implement the change suggested.

d. I think the paper would benefit from more introduction and framing of the exploratory results section. I would like to see some framing in the introduction that prepares the reader for these results, introduces the concepts of interoceptive awareness, decentering, and reasons for meditating and why they might be important for the research questions.

In light of suggestions to keep the introduction well structured, we added a whole section on these matters in the supplementary introduction. In paragraph six of the introduction (Page 4) we have included something about interoception and decentring:

We also measured self-reports of cognitive processes previously suggested as core mechanisms of the beneficial impacts of mindfulness interventions^37^. Specifically, we measured interoception (ability to have sensory awareness of the bodily state)^38^ and decentring (ability to observe one’s thoughts and feelings objectively)^39^.

e. The limitations section is quite short and could include quite a lot more. I’d like to see more limitations about the sample (how accurately does it generalize to a larger population), the analyses used, the measures used, and the theory that distinguishes mystical experiences from psychosis. Finally, I’d like to see a conclusion section after the limitations.

This has been added.
