# Supplementary material for: Differential relationship between meditation methods and psychotic-like and mystical experiences
Source: PLoS One. 2024 Dec 5;19(12):e0309357. doi: 10.1371/journal.pone.0309357 (PMC11620446; doi:10.1371/journal.pone.0309357)
Supplement: Attachment — Submitted filename: Reviewer Comments 6.docx [file pone.0309357.s004.docx]

Reviewer #1: All my comments have been addressed, thank you. I look forward to seeing this manuscript published as it will be an interesting contribution to the field.

We thank reviewer 1 for their suggested edits and additions, we believe this has improved the manuscript. We have been careful not to edit or remove any of the previous changes suggested by reviewer 1 in the latest round of edits. Some of the previous changes that we added to the supplements may now be in the main text, as this is one of the biggest changes made.

Reviewer #2: Thank you for the opportunity to review this interesting manuscript. This revision represents a significant improvement over the previous draft. The authors have clearly put a lot of work into responding to the comments from the previous round of revisions. Many of the technical and theoretical issues have now been sufficiently addressed in my opinion. Furthermore, the results of this study are interesting, important, and novel. I believe that the study does merit publication. Unfortunately, however, I still think it needs significant work in the following areas before it is ready for publication:

**Writing quality:** the manuscript needs a great deal of further proofreading and formatting.

We thank the reviewer for his kind words of encouragement, and acknowledgement of work put into previous edits. We have carefully read each comment and done our best to fully address each one appropriately. Large edits have been applied to the introduction and discussion, with the addition of a lot more discussion of background work and relevant concepts. We believe this has improved the article and given it more depth, so we thank the reviewer for their suggestions. For clarity, we have removed sections from the ‘supplements’ including parts of the supplementary intro, results and methods. This is because much of this is now included in the main text in response to some of the requests below.

More generally we have worked to correct grammar, spelling and punctuation. We have adjusted the method section in line with suggestions. Each comment has a reply to outline what we have done and where to find the alterations which are highlighted in red in the manuscript.

1. There are still grammatical issues, run-on sentences, and spelling mistakes throughout the manuscript. For example, decentering is spelled incorrectly on second page of introduction, “and” is spelled incorrectly in the title of Table 2, capitalization is inconsistent, etc.

We have addressed this.

2. The manuscript needs additional formatting according to APA style or other journal standards. For example, sentences should be in past tense, tables and figures should be referenced and contextualized in the text, and headers should be structured in a more standard way with main headers and subheaders.

We have done this.

3. Acronyms should be introduced first with the full name (e.g., CAPE-42 in the introduction).

We have done this.

4. The language in many places needs to be clearer and more precise. For example, please clarify differences between “related to, linked to, defined as, correlated with” which seem to be used interchangeably at times (especially in second page of introduction). Describing how a construct is defined should be clearly delineated from describing what it is correlated with.

We have reduced the number of terms referencing the same thing. We have edited grammar etc.

5. The study should not repeat so many times that it is pre-registered. This can be mentioned in the methods once. Also, avoid language like “a very large sample” and instead just state the size of the sample.

We have removed extra instances of pre-registered and very large sample is gone.

Introduction:
1. I feel that the introduction still needs some work as it is too focused on describing the present study rather than introducing the concepts and theory necessary to contextualize the present study. There are a number of sentences in the introduction that would be better placed in the methods or results. For example on page 1, the following sentence describes the study method and should not be in the introduction: “Here, we examined the impact of different types of meditation methods on PLEs. To allow statistical tests, we grouped them according to a taxonomy proposed by Nash, Newberg and Awasthi.” The last sentence in the introduction is about the results and should be moved to that section.

We removed suggested sentences. And we expanded on discussion of background work.

2. The introduction needs to introduce and describe the concepts more clearly. For example, what is an enhanced cognitive state? Why are MBSR and MBCT considered CDM meditation (and how do you know this since these interventions involve multiple techniques)?

We have expanded on definitions of MBCT and MBSR and why we consider them CDM. This links to the expanded description of meditation taxonomies and how we used them.

3. The first paragraph of the second page about reasons to meditate provides little theory or context and instead focuses on the study’s research questions. Why are reasons for meditating important? What do other studies show about reasons to meditate?

We have added more about reasons to meditate and their importance.

4. Given the importance placed in Table 2 on the three different meditation taxonomies, I think these need to be described in more detail in the introduction in order to provide more context for the rest of the paper.

A more detailed discussion of these taxonomies has been added.

5. The concepts of decentering and interception are very briefly mentioned and also need to be further defined. Why would these core mechanisms possibly relate to PLEs? Why are these considered core mechanisms?

This has been added.

6. I think the introduction could say something more about the effects of meditation on altered states. The framing of the first paragraph says that the study looks at the links between different types of meditation and adverse and beneficial outcomes. While this is technically correct, it feels a bit odd not to mention that both of these outcomes (PLEs and mystical experiences) are types of altered states. In general, I think the introduction could benefit from a more in-depth literature review, which would also improve the quality of the discussion.

We have made considerable changes to the introduction, including discussion of altered states.

Methods:
1. Analyses section of methods should be under a single header with subheaders for the various types of analyses.

We have done this.

2. Questionnaires should be a subheader under materials and methods. Techniques, traditions, reasons for meditating, and each individual questionnaire don’t need separate headers.

We have done this.

3. Questionnaire descriptions are awkwardly written and formatted

These have been edited.

4. Please include a bit more about the recruitment strategy. How was the study advertised? This has an impact on the sample characteristics and should also be referenced in the limitations (about how the sample might not generalize to all people).

We have included some more detail in the methods section ‘Procedure’ and added to the limitations section.

Results:
1. Demographics: what about race, ethnicity, nationality, first-language, sexual orientation?

We did not collect this information. But we have added this to the limitations section.

2. I still feel that the tables and figures need to be better referenced in the text and more clearly described. Table 2 contains useful information but it presents so many different categorical systems that it took me some time to make sense of. I think this table in particular needs to be better described in the text.

This table has now been described in some detail in the main text.

Discussion:
1. The discussion doesn’t mention anything about interoception or decentering. I think these should be discussed since they’re part of the results.

This has been added in some detail under the title ‘Decentering and interoceptive awareness’.

2. In general, the discussion could use more integration with the other literature and theoretical considerations instead of just a summary of the results. Why is body focus important? Why is null focus important? Why do you think reasons for meditating are important?

We have added a discussion of the ‘null’ element of NDM and body focus in the discussion under the section titled ‘NDM techniques and low body focus correlate with PLEs’. We added to the discussion about reasons to meditate in the discussion section: ‘Meditating for spiritual reasons correlates with PLEs’.

We have added further subtitles in the discussion to make it clearer.

7. PLOS authors have the option to publish the peer review history of their article ([what does this mean?](https://journals.plos.org/plosone/s/editorial-and-peer-review-process#loc-peer-review-history)). If published, this will include your full peer review and any attached files.


If you choose “no”, your identity will remain anonymous but your review may still be made public.


**Do you want your identity to be public for this peer review?** For information about this choice, including consent withdrawal, please see our [Privacy Policy](https://www.plos.org/privacy-policy).

Reviewer #1: No

Reviewer #2: **Yes:** Nicholas K Canby
